# Therapeutic silencing of lncRNA RMST alleviates cardiac fibrosis and improves heart function after myocardial infarction in mice and swine

**DOI:** 10.7150/thno.82543

**Published:** 2023-06-26

**Authors:** Teng Ma, Fan Qiu, Yanshan Gong, Hao Cao, Gonghua Dai, Daohan Sun, Dongling Zhu, Han Lei, Zhongmin Liu, Ling Gao

**Affiliations:** 1 Translational Medical Center for Stem Cell Therapy & Institute for Regenerative Medicine, Shanghai East Hospital, Tongji University School of Medicine, Shanghai 200123, China.; 2 Department of Thoracic Cardiovascular Surgery, The Eighth Affiliated Hospital of Sun Yat-sen University, Shenzhen, Guangdong 518033, China.; 3 Department of Cardiovascular and Thoracic Surgery, Shanghai East Hospital, Tongji University School of Medicine, Shanghai 200120, China.; 4 Department of Radiology, Shanghai East Hospital, Tongji University School of Medicine, Shanghai 200120, China.; 5 The First Affiliated Hospital of Jinzhou Medical University, Jinzhou, Liaoning 121001, China.; 6 Department of Respiratory Medicine, Shanghai East Hospital, Tongji University School of Medicine, Shanghai 200120, China.; 7 Shanghai Institute of Stem Cell Research and Clinical Translation, Shanghai East Hospital, Tongji University, Shanghai 200120, China.

**Keywords:** cardiac fibrosis, lncRNA RMST, miR-24-3p, lysyl oxidase, pig

## Abstract

**Rationale:** Cardiac fibrosis is an adverse consequence of aberrant fibroblast activation and extracellular matrix (ECM) deposition following myocardial infarction (MI). Recently, long noncoding RNAs (lncRNAs) have been reported to participate in multiple cardiac diseases. However, the biological functions of lncRNA rhabdomyosarcoma 2-associated transcript (RMST) in cardiac fibrosis remain largely unknown.

**Methods:** The role of RMST in regulating cardiac fibroblast (CF) proliferation, fibroblast-to-myofibroblast transition (FMT), and ECM production, which were induced by transforming growth factor-β1, was evaluated through immunofluorescence staining, cell contraction assay, cell migration assay, qRT-PCR, and western blot. The therapeutic effect of RMST silencing was assessed in murine and porcine MI models.

**Results:** The present study showed that RMST expression was upregulated and associated with cardiac fibrosis in murine and porcine MI models. Further loss-of-function studies demonstrated that RMST silencing in vitro significantly inhibited CF proliferation, FMT, and ECM production. Accordingly, RMST knockdown in vivo alleviated cardiac fibrosis and improved cardiac contractile function in MI mice. Moreover, RMST acted as a competitive endogenous RNA of miR-24-3p. miR-24-3p inhibition abolished, while miR-24-3p agomir reproduced, the RMST knockdown-mediated effects on CF fibrosis by regulating the lysyl oxidase signaling pathway. Finally, the therapeutic potential of RMST knockdown was evaluated in a porcine MI model, and local RMST knockdown significantly inhibited cardiac fibrosis and improved myocardial contractile function in pigs after MI.

**Conclusion:** Our findings identified RMST as a crucial regulator of cardiac fibrosis, and targeting RMST may develop a novel and efficient therapeutic strategy for treating fibrosis-related cardiac diseases.

## Introduction

Myocardial infarction (MI), which typically leads to massive loss of cardiomyocytes and pathological cardiac remodeling, remains the leading cause of death and economic burden globally 1. Therefore, it is of great significance to investigate the underlying mechanisms and develop novel therapeutic strategies. Cardiac fibrosis is characterized by aberrant fibroblast proliferation, fibroblast-to-myofibroblast transition (FMT), and excessive extracellular matrix (ECM) deposition 2, 3. Cardiac fibrosis initially occurs as a compensatory response to maintain the structural and functional integrity of the injured heart. However, excess fibrosis of the infarct area results in heart systolic and diastolic dysfunction, arrhythmia, and even increased occurrence of sudden cardiac death 4, 5. There is accumulating evidence that cardiac fibroblasts (CFs) are the predominant orchestrators of fibrotic responses following MI 6, 7. CFs maintain a quiescent phenotype in physiological conditions, rarely undergoing mitosis and secreting ECM. Under pathological conditions, CF proliferation and FMT are the early events in the process of excessive ECM production and collagen-rich fibrotic scar formation 8, 9. However, the molecular mechanism of FMT following MI remains largely unknown. Therefore, finding the initiating signals that play an essential role in the process of cardiac fibrosis is crucial for developing novel therapeutic strategies for ischemic heart disease.

Long noncoding RNAs (lncRNAs), which are more than 200 nucleotides in length, participate in multiple physiological and pathological processes 10, 11. Although the role of lncRNAs in the development of some cardiovascular disorders has been documented, the functions of lncRNAs in cardiac fibrosis, especially in FMT, are not yet understood. Therefore, further study of the potential mechanisms of lncRNAs in pathological cardiac remodeling may facilitate the development of new strategies for treating MI. Recently, lncRNA rhabdomyosarcoma 2-associated transcript (lncRNA RMST) has been demonstrated to participate in various human diseases, including cancer, stroke, and parkinsonism 12-14. However, the functions of RMST in cardiac fibrosis have not been sufficiently investigated.

A large amount of lncRNA-centered research illustrates that lncRNAs can regulate transcription, translation, post-translational modifications, and epigenetic modifications by interacting with DNA or RNAs 15, 16. Interestingly, although nuclear lncRNAs are more abundant, they are not as stable as cytoplasmic lncRNAs. In the cytoplasm, lncRNAs mainly exert biological functions through the sponge mechanism 17. lncRNAs can sequester miRNAs and proteins to regulate their activity and levels, influence protein post-translational modifications, or mediate mRNA translation and stability 18. However, the pathway through which RMST plays a regulatory role in cardiac fibrosis needs to be explored.

Due to the vast differences in cardiac and coronary anatomy and hemodynamics between rodents and humans, experimental data obtained from small animal models provided limited reference information for clinical translation 19. Therefore, mammalian (e.g., pig and macaques) models have more pivotal translational value for cardiovascular disease research. Here, we investigated whether RMST silencing could reverse cardiac fibrosis and recover damaged heart function after MI in pigs. To the best of our knowledge, this is the first study focusing on the therapeutic effect of lncRNA in a preclinical porcine MI model.

In the current study, we aimed to identify the novel function of RMST in regulating cardiac fibrosis. To address this, we performed a series of gain- and loss-of-function experiments to investigate the effects of RMST on CF proliferation and FMT in vitro. We also evaluated the therapeutic effect of RMST silencing in female murine and porcine MI models.

## Materials and Methods

### Ethics statement

All experimental procedures, including animal studies, were approved by the Institutional Animal Care and Use Committee of Tongji University. All animal experiments complied with the NIH Guide for the Care and Use of Laboratory Animals.

### CF culture and chemical treatments

Mouse CFs were purchased (Procell, Wuhan, China) and maintained in Dulbecco's Modified Eagle Medium Nutrient Mixture F-12 (DMEM/F12, ThermoFisher Scientific, CA, USA) supplemented with 10% fetal bovine serum (FBS) at 37 °C. CFs were passaged using 0.5% trypsin (ThermoFisher Scientific, CA, USA) when cells were grown to 80% confluence.

For RMST silencing, we used a lncRNA Smart Silencer System (RiboBio, Guangzhou, China) following the manufacturer's instructions. Briefly, CFs (2 × 10^5^ cells/well) were seeded in 6-well plates and transfected with RMST Smart Silencer (1-50 nM) using Lipo3000 reagent (ThermoFisher Scientific, CA, USA). For RMST overexpression, the RMST overexpression plasmid pcDNA3.1-RMST was constructed (Hanbio, Shanghai, China) and transfected into cultured CFs using Lipo3000 reagent (ThermoFisher Scientific). An empty pcDNA3.1 plasmid (vector) was used as a control. For miR-24-3p loss- or gain-of-function experiments, CFs (2 × 10^5^ cells/well) were seeded in 6-well plates and transfected with 10 nM agomiR-24-3p, 20 nM antagomiR-24-3p, or 10 nM negative control (NC) with Lipo3000 following the manufacturer's instruction. For lysyl oxidase (LOX) gain-of-function experiments, lentiviruses overexpressing LOX (Lenti-LOX, VB900004-4690bgv, VectorBuilder, Shanghai, China) were used in line with the manufacturer's instructions, and 1 x 10^6^ transducing units (TU) lentivirus were added into CFs (2 × 10^5^ cells/well). For FMT, CFs were treated with 10 ng/mL recombinant human TGF-β1 (R&D system, CA, USA) for 72 h.

### Cell proliferation analysis

CF proliferation analysis was performed using Ki67 immunofluorescence staining, as reported previously 6. Briefly, cultured cells were fixed with 4% paraformaldehyde and permeabilized with 0.2% Triton X-100. Then, Ki67 antibody was used to label the cells in mitosis, and vimentin antibody was used to identify fibroblasts. Ki67-positive fibroblasts were photographed, and the number of these cells was calculated from at least five random visual fields per sample.

### Cell migration assessment

Cell migration assay was performed as described in a previous study 20. Briefly, CFs (2 × 10^5^ cells/well) were cultured in 6-well plates and induced with 10 ng/mL TGF-β1 for 48 h; then, a 10-μL pipette was used to generate artificial wounds; and cells were labeled with calcein and cultured with DMEM/F12 supplemented with 10% FBS and 10 ng/mL TGF-β1 for another 24 h. The average extent of wound closure was quantified.

### Cell contractile activity analysis

Cultured CFs were treated with 10 ng/mL TGF-β1 for 24 h and then harvested. 4 × 10^6^ isolated CFs were added to 750 μL fibronectin solution in 6-well plates; then, 750 μL thrombin solution (20 U/mL, R&D system, CA, USA) was added into the same wells and incubated at 37 °C for 5 min for polymerization. After polymerization, the fibrin scaffold was transferred to new 6-well plates and cultured with DMEM/F12 supplemented with 10% FBS, 1% 6-aminocaproic acid (Sangon Biotech, Shanghai, China), and 10 ng/mL TGF-β1. The diameter changes of the fibrin scaffold were calculated after 48 h.

### Cardiac cell isolation

For cardiomyocyte isolation, mouse hearts were digested with type II collagenase using a Langendorff-free system as previously described 21. After sufficient digestion, the cell suspension was transferred to an upright falcon tube; 10 min later, the cardiomyocytes that settled to the tube's bottom through gravity were collected. For endothelial cell isolation, the cell suspension was collected via centrifugation, and endothelial cells were isolated using CD31-coupled microbeads 22. For CF isolation, the cells were plated onto collagen-coated soft hydrogel-bound polystyrene plates and incubated in a humidified incubator for 150 min. Then, the unattached cells and debris were removed to obtain CFs 23.

### mRNA profiling

RMST knockdown CFs and controls were prepared for RNA sequencing (RNA seq). RNA-seq experiments were performed by Lianchuan Biotech Co., Ltd. (Hangzhou, China) in line with a standard procedure. Analysis of differential expression was performed using the Lianchuan Biological Cloud Platform.

### Fluorescence in situ hybridization (FISH)

Localization of RMST in CFs was detected using a lncRNA FISH Probe Mix kit (RiboBio, Guangzhou, China) following the manufacturer's protocol. Briefly, cells (2 × 10^4^ cells/well) were seeded in a 4-chamber Lab-Tek II Chamber Slide and cultured in DMEM/F12 supplemented with 10% FBS for 24 h. Next, the cells were fixed with 4% paraformaldehyde at room temperature. Then, the fixed cells were treated with pre-cooled Triton X-100 (0.5% dissolved in 1× PBS). After washing with 1× PBS, the cells were incubated overnight with a 20 µM Cy3-conjugated FISH probe in the hybridization buffer (100 μL).

### Luciferase reporter assay

Luciferase reporter assay was performed as described in a previous study 24. In brief, CFs (5 × 10^4^ cells/well) were seeded in 24-well plates and co-transfected with RMST luciferase reporter plasmids and agomiR-24-3p or miR-24-3p negative control (NC) using Lipo3000 as described above. After 48 h, the luciferase activity was detected by a dual-luciferase reporter (DLR) assay system (Promega, WI, USA). Renilla luciferase activity was used as an internal control.

### Animal studies

C57BL/6 mice (female, 20 g, 8-12 weeks old) were purchased from Shanghai SLAC Laboratories Co., Ltd. The mice were anesthetized with 2% isoflurane and ventilated using a small animal ventilator (ALC-Bio, Shanghai, China). The mouse hearts were exposed by cutting open the fourth intercostal space. The left anterior descending artery (LAD) was ligated 2 mm from the root with an 8-0 nonabsorbable thread. For RMST knockdown, we introduced RMST-specific shRNA (**Table S1**) into the mouse hearts using an adeno-associated virus serotype 6 (AAV6)-mediated delivery system (HanBio, Shanghai, China). Specifically, 1 × 10^11^ viral genome (vg) particles of AAV6 vectors (10 µL) carrying scrambled control shRNA (MI+RMST NC group) or RMST-specific shRNA (MI+RMST Kd group) were injected into the infarct and the peri-infarct zones of the hearts with MI. Mice in the sham group underwent all surgical procedures for MI induction, except for occlusion, and recovered without any experimental treatments.

Bama pigs (female, 20 kg, 16-20 weeks old) were purchased from Shanghai Jiagan Biotech Co., Ltd. (China). The pigs were anesthetized with 2% isoflurane and ventilated using an anesthesia ventilator (MATRX, MD, USA). The pig hearts were exposed by the left thoracotomy on the fourth intercostal space. The first and the second diagonal coronary arteries from the LAD were occluded at the root with 4-0 suture for 1 h and then re-perfused. AAV6 vectors 25 (1 mL, 1 × 10^13^ vg particles) carrying scrambled control shRNA (MI+RMST NC group) or RMST-specific shRNA (MI+RMST Kd group) (**Table S1**) were injected into the infarct and the peri-infarct zones of the hearts with MI at the onset of reperfusion. Animals in the sham group underwent all surgical procedures except occlusion and recovered without any experimental treatments. Animals in all treatment groups received standard postoperative care, including analgesia and antibiotic administration, until they ate normally and became active.

### Cardiac function analysis

Echocardiography was performed using an MS-400 transducer (30 Hz, Visual Sonics, Toronto, Canada) and a Vevo2100 system (Visual Sonics, Toronto, Canada) to evaluate the murine cardiac function as described previously 26. The mice were anesthetized with 2% isoflurane, and the heart rate was controlled at 400 beats/min. Both conventional two-dimensional and M-mode images of the heart were acquired in a parasternal short-axis view. Vevo Analysis software was used to calculate left ventricular ejection fraction (LVEF) and fractional shortening (LVFS). For pigs, cardiac function was assessed by high-resolution computed tomography (CT, Siemens, MUC, Germany). Briefly, the pigs were anesthetized with 2% isoflurane and placed in the supine position. Before CT scanning, an iodinated contrast agent (0.7 mL/kg, Abcam, CB, UK) was injected (3 mL/s) through the ear vein. Scanning was conducted using a prospective ECG-triggered dynamic dual-energy acquisition protocol. The hearts were scanned in an approximate horizontal long axis, and 20 axial scans were obtained for every heartbeat. End-systolic and end-diastolic data sets based on the smallest and largest intra-ventricular areas at a mid-ventricular location, respectively, were reconstructed for each CT study. Infarct size was measured via delayed enhancement CT.

### Hemodynamic measurement

A hemodynamic monitoring system (LabChart, AD Instruments, Shanghai, China) was used to evaluate the LV function as previously reported 27. An invasive monitor was introduced percutaneously through the left carotid artery and aorta into the LV lumen under continuous monitoring of the pressure curves until the diastolic pressure indicated localization in the LV.

### Quantitative real-time PCR (qRT-PCR)

qRT-PCR was performed as described in a previous study 20. The total RNA was isolated from cell and tissue samples using TRIzol reagent. The cDNA synthesis was performed using the PrimeScript RT reagent kit and the PrimeScript miRNA cDNA Synthesis kit (TaKaRa, Dalian, China). qRT-PCR was performed using the SYBR Premix Ex Taq II kit (TaKaRa) with an ABI 7500 qPCR instrument (ThermoFisher Scientific). U6 and GAPDH were used as internal controls. The relative expression of miRNA or mRNAs was evaluated by the 2^-ΔΔCt^ method. The primer sequences are provided in **Table S2**.

### Western blot (WB)

WB was performed as described in a previous study 28. Cell and tissue samples were lysed with RIPA supplemented with a protease inhibitor cocktail (MedChem Express, Shanghai, China). Equal amounts of protein were fractionated on a 6-15% SDS-PAGE and then transferred to PVDF membranes (Millipore, MA, USA). The PVDF membranes were probed with primary antibodies of interest (**Table S3**), and β-tubulin or GAPDH was used as an internal control. The protein bands were detected using enhanced chemiluminescence (ECL) reagent (Cell Signaling Technology, MA, USA). The intensities of the bands were quantified by ImageJ software.

### Histology and immunofluorescence staining

Histology and immunofluorescence staining were performed as described in a previous study 29. The heart tissue samples were collected and embedded in an optimal cutting temperature compound (OCT) for serial cryosectioning. The sections were stained with Masson or hematoxylin and eosin (H&E) for histology analysis in line with the manufacturer's instructions. For triphenyl tetrazolium chloride (TTC) staining, the heart tissue samples were excised and cut into 2-mm slices perpendicular to the LAD from the apex to the base. Then, the slices were immersed in 1% TTC solution at 37 °C for 20 min to visualize the infarct size 30. Immunofluorescent staining in the heart tissue sections was performed, as previously described 31. Briefly, the heart sections were fixed with 4% paraformaldehyde, permeabilized in 0.25% Triton X-100, blocked in Ultra-V Block buffer, incubated overnight at 4 °C with primary antibodies, and then incubated for 2 h at room temperature with the corresponding secondary antibodies. For wheat germ agglutinin (WGA) staining, the sections were stained for 1 h at room temperature using an FITC-labeled WGA dye (Sigma-Aldrich, MO, USA).

### Statistical analysis

Data are presented as mean ± SEM. Comparison between the two groups was performed using the two-tailed unpaired Student's *t* test. Comparison between three or more groups was performed using one-way analysis of variance (ANOVA) followed by post-hoc Bonferroni test. Survival analysis was performed with the log-rank test. Statistically significant difference was considered when *P* < 0.05. Statistical analyses were performed using GraphPad Prism 9.0 (GraphPad Software, CA, USA).

## Results

### RMST expression is upregulated in the murine and porcine hearts after MI

**Figure 1:**
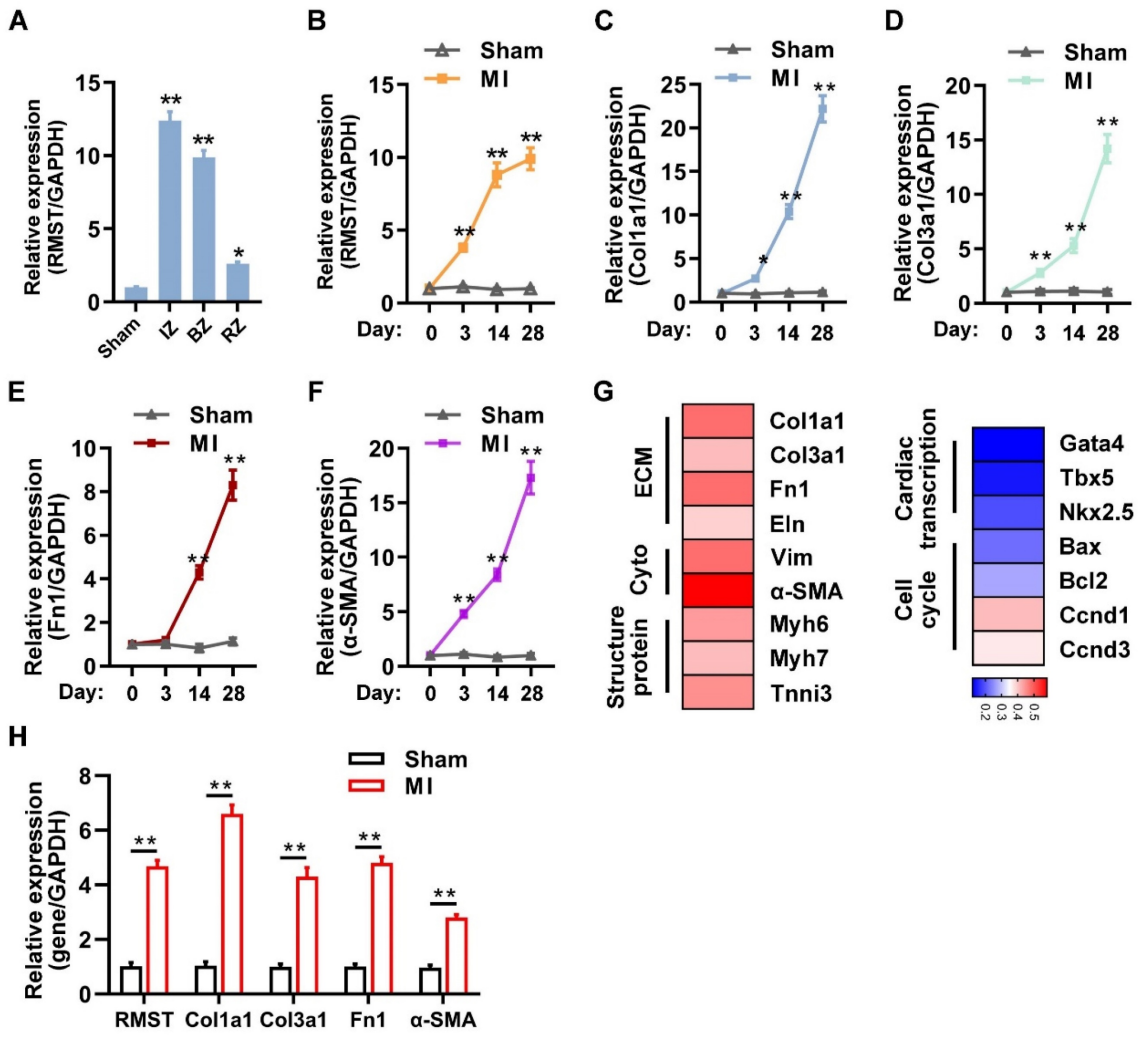
**lncRNA RMST expression is upregulated in the murine and porcine hearts after myocardial infarction (MI)**.** (A)** The mRNA expression of RMST in the infarct zone (IZ), border zone (BZ), and remote zone (RZ) of infarcted mouse hearts was detected by qRT-PCR 28 days after MI. Sham hearts served as a control. *n* = 4 in each group. **(B-F)** The mRNA expression of (**B**) RMST, **(C)** Col1a1, **(D)** Col3a1, **(E)** fibronectin 1**,** and **(F)** α-SMA in the BZ of infarcted mouse hearts or the corresponding zone of the sham hearts was detected by qRT-PCR. *n* = 4-8 in each group.** (G)** The mRNA expression of protein-coding genes related to the extracellular matrix (ECM), cytoplasm (Cyto), structural protein, cardiac transcription, and cell cycle in the border zone (BZ) of infarcted mouse hearts was detected by qRT-PCR 28 days after MI. A heat map of mRNA expression correlations between RMST and these genes was generated. **(H)** The mRNA expression of RMST, Col1a1, Col3a1, fibronectin 1, and α-SMA in the BZ of infarcted porcine hearts or the corresponding zone of the sham hearts was detected by qRT-PCR 28 days after MI (*n* = 4 in each group). Significance was evaluated via Student's *t* test. *P < 0.05, **P < 0.01 vs. values in the sham group or as indicated on the panels. lncRNA, long noncoding RNA; RMST, rhabdomyosarcoma 2-associated transcript; Col1a1, collagen type I alpha 1; Col3a1, collagen type III alpha 1; Fn1, fibronectin 1; Eln, elastin; Vim, vimentin; α-SMA, alpha-smooth muscle actin; Myh6, myosin heavy chain 6; Myh7, myosin heavy chain 7; Tnni3, troponin I3; Gata4, GATA binding protein 4; Tbx5, T-Box transcription factor 5; NKX2.5, NK2 homeobox 5; Bax, Bcl2-associated X; Bcl2, B-cell lymphoma-2; Ccnd1, cyclin D1; and Ccnd3, cyclin D3.

We first investigated the expression and association between RMST and fibrotic genes in the murine model of MI. TTC staining showed that sizable infarct areas in the mouse hearts had been developed on day 3 after MI (**Figure S1A-B**). Masson and H&E staining showed that large fibrous scars and extensive collagen deposition had formed in the infarcted area on day 28 after MI (**Figure S1C-D**). LVEF and LVFS in the MI group assessed by echocardiography on day 28 after MI markedly decreased compared with those in the sham group (**Figure S1E-G**). The qRT-PCR results showed that the mRNA expression of RMST was significantly upregulated in the infarct zone (IZ), border zone (BZ), and remote zone (RZ) of MI hearts 28 days after MI, specifically in the IZ and BZ, compared to sham ones (**Figure 1A**), and the mRNA expression of RMST sharply increased in CFs, slightly increased in cardiomyocytes, and did not change in endothelial cells in murine hearts on day 28 after MI (**Figure S2A-C**). Additionally, with the development of MI, the mRNA expression of RMST and fibrotic genes, including collagen type I alpha 1 (Col1a1), collagen type III alpha 1 (Col3a1), alpha-smooth muscle actin (α-SMA), and fibronectin 1, was progressively upregulated in different phases of cardiac remodeling in the BZ of MI hearts compared with sham hearts (**Figure 1B-F**), indicating that RMST expression may be related to fibrotic progress. A heat map was generated to examine the correlation of RMST and protein-coding genes (PCGs) related to ECM, cytoskeleton, structural proteins, cardiac transcript factors, and the cell cycle; the results showed that RMST expression was highly related to ECM, cytoskeleton, and structural proteins, but not to cardiac transcript factors (**Figure 1G**). Furthermore, the assessments of mRNA expression of RMST and fibrotic genes in the BZ of the infarcted porcine hearts 28 days after MI showed that RMST expression significantly increased in the infarcted hearts compared with the sham group, which was in line with the changes in the fibrotic genes, including Col1a1, Col3a1, fibronectin 1, and α-SMA (**Figure 1H**). Overall, these results demonstrate that the expression of RMST increases after MI and may participate in cardiac fibrosis through regulating CFs.

### RMST knockdown mitigates TGF-β1-induced CF fibrogenesis in vitro

**Figure 2:**
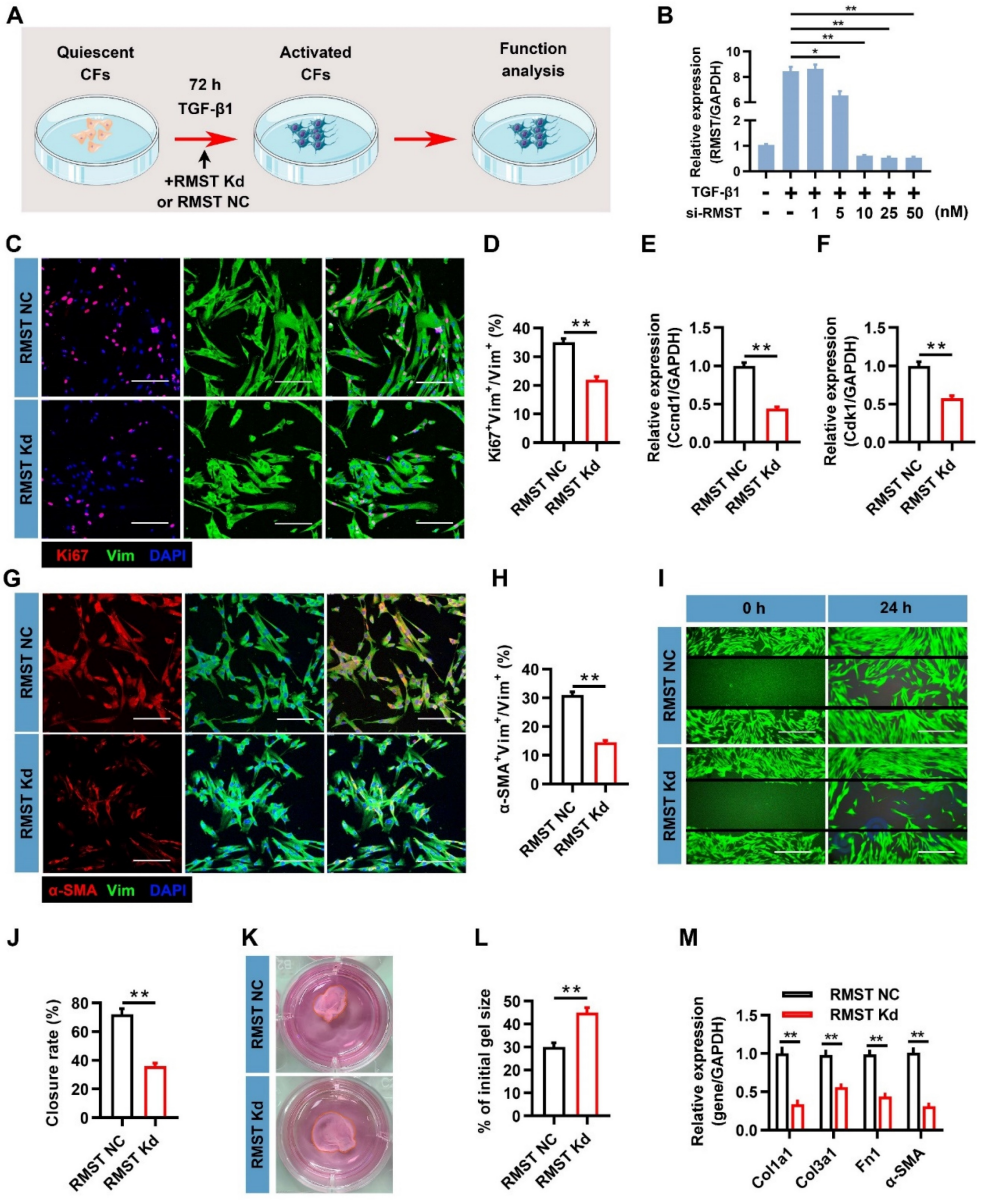
**RMST knockdown alleviates TGF-β1-induced cardiac fibroblast (CF) fibrogenesis. (A)** Workflow: CFs were treated with RMST negative control (NC) or RMST silencer for knockdown (Kd) in the presence of TGF-β1 for 3 days, and subsequent in vitro analyses were performed. **(B)** RMST mRNA expression in CFs transfected with RMST smart silencer at increasing concentrations (1-50 nM) was detected by qRT-PCR. RMST smart silencer concentration of 10 nM was finally selected for subsequent experiments.** (C)** TGF-β1-treated CFs were immunofluorescently stained for the expression of vimentin (fibroblast marker) and Ki67 (cell proliferation marker), and nuclei were counterstained with DAPI. Scale bar = 100 μm.** (D)** Quantification of Ki67-positive cells in CFs.** (E-F)** The mRNA expression of **(E)** Ccnd1 and **(F)** Cdk1 in CFs treated with TGF-β1 was detected by qRT-PCR. **(G)** TGF-β1-treated CFs were immunofluorescently stained for α-SMA (FMT indicator) and vimentin expression, and nuclei were counterstained with DAPI. Scale bar = 100 μm. **(H)** Quantification of α-SMA-positive cells in CFs. **(I)** Wound healing assay assessed the migration ability of TGF-β1-treated CFs. Scale bar = 200 μm. **(J)** Quantification of closure rate. **(K)** Shrink ring assay assessed the contractility ability of TGF-β1-treated CFs.** (L)** Quantification of fibrin scaffold contraction.** (M)** The mRNA expression of Col1a1, Col3a1, fibronectin 1, and α-SMA in TGF-β1-treated CFs was detected by qRT-PCR. *n* = 4 to 5 independent experiments. Significance was evaluated via Student's *t* test (D-F, H, J, L, and M) and one-way ANOVA with the post-hoc Bonferroni test (B). *P < 0.05 and **P < 0.01. Vim, vimentin; Fn1, fibronectin 1; Ccnd1, cyclin D1; Cdk1, cyclin-dependent kinase 1; Col1a1, collagen type I alpha 1; and Col3a1, collagen type III alpha 1.

To characterize the function of RMST in the course of TGF-β1-induced CF fibrogenesis, a loss-of-function approach was applied in CFs through transfection with lncRNA Smart Silencer, which downregulated RMST expression (**Figure 2A**). A concentration-dependent assay showed that 10 nM of Smart Silencer was sufficient to achieve maximal RMST knockdown in CFs (**Figure 2B**), and this concentration was selected to perform subsequent experiments. CFs proliferated massively during the fibrogenesis progress; however, RMST knockdown markedly inhibited CF proliferation, as determined by Ki67 fluorescent immunostaining (**Figure 2C-D**). qRT-PCR results also showed that RMST knockdown significantly downregulated the mRNA (**Figure 2E-F**) and protein (**Figure S3A-B**) expression of proliferation-related genes, such as cyclin D1 (Ccnd1) and cyclin-dependent kinases 1 (Cdk1). Furthermore, immunofluorescence staining assessment showed that RMST knockdown blocked TGF-β1-induced FMT, as evidenced by a reduction in the number of α-SMA^+^ CFs (**Figure 2G-H**) and Ki67^+^/α-SMA^+^ CFs (**Figure S3C-D**). Migration and contractile capacities are crucial for myofibroblasts to maintain the differentiated phenotype, and they can be evaluated by wound healing assay and shrink ring assay. The decreased wound closure rate (**Figure 2I-J**) and fibrin scaffold contraction (**Figure 2K-L**) in the RMST knockdown group compared with the control group revealed that RMST silencing was able to attenuate the migration and contractile capacities of activated fibroblasts, which also indicated reduced FMT. Finally, the qRT-PCR results showed that RMST knockdown significantly downregulated the mRNA expression of the fibrotic genes, including Col1a1, Col3a1, fibronectin 1, and α-SMA, in TGF-β1-treated CFs (**Figure 2M**), which was in line with the change in Col1a1 and fibronectin 1 protein expression (**Figure S3E-F**). Overall, these results demonstrate that RMST knockdown significantly relieves TGF-β1-induced CF fibrogenesis.

### RMST regulates CF gene programs

To better investigate the effect of RMST silencing on regulating gene programs during CF activation, we performed RNA-seq analysis on control and RMST knockdown CFs in the presence of TGF-β1. With the selection criteria fold change > 2 and adjusted *P* value < 0.05, a total of 3541 differentially expressed genes (DEGs, 1654 upregulated, 1887 downregulated) were identified, and a heat map and a volcano map of the DEGs were generated (**Figure S4A-C**). Further Gene Ontology (GO) term enrichment analysis illustrated that RMST knockdown affected the cytoplasm, ECM, and collagen binding (**Figure S4D**). Similarly, KEGG pathway analysis indicated that the DEGs were related to ECM-receptor interaction, focal adhesion, and the PI3K-Akt signaling pathway (**Figure S4E**). Overall, these results indicate that RMST plays a vital role in regulating fibrogenesis.

### RMST knockdown alleviates cardiac fibrosis and improves cardiac contractile function in a murine MI model

**Figure 3:**
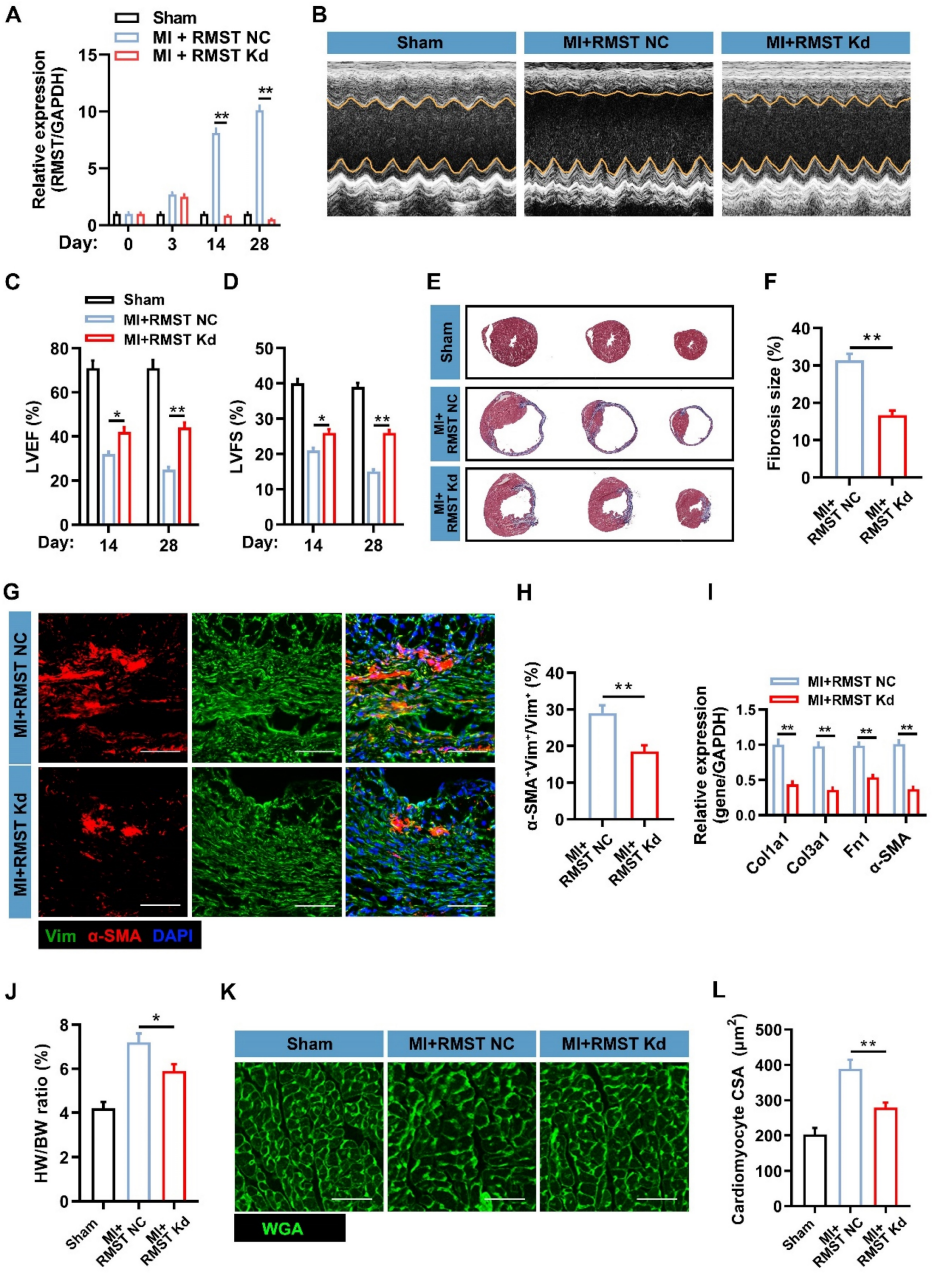
** RMST knockdown alleviates cardiac fibrosis and improves cardiac function in a murine MI model**. The mice were divided into the sham group, the MI+RMST NC group (after MI surgery, animals received 10 μL of AAV6 vectors carrying scrambled shRNA), and the MI+RMST Kd group (after MI surgery, animals received 10 μL of adeno-associated virus serotype 6 (AAV6) vectors carrying RMST-specific shRNA. **(A)** The mRNA expression of RMST was detected by qRT-PCR on days 0, 3, 14, and 28 after MI. *n* = 4-8 in each group.** (B)** The representative images of echocardiography on day 28 after MI or sham surgery.** (C-D)** Quantification of **(C)** LVEF and **(D)** LV fractional shortening (LVFS) measured by echocardiography on days 14 and 28 after MI or sham surgery. *n* = 8-10 in each group.** (E)** Fibrosis size was detected by Masson staining on day 28 after MI. **(F)** Quantification of fibrosis size. **(G)** Sections from the BZ of infarcted hearts were immunofluorescently stained for the expression of α-SMA and vimentin on day 28 after MI. Scale bar = 200 μm. **(H)** Quantification of α-SMA fluorescence intensity in fibroblasts. **(I)** The mRNA expression of Col1a1, Col3a1, α-SMA, and fibronectin 1 in the BZ of infarcted hearts was detected by qRT-PCR. **(J)** The heart weight/body weight (HW/BW) ratio was measured on day 28 after MI. **(K)** Sections from the BZ of infarcted hearts were stained with wheat germ agglutinin (WGA) on day 28 after MI. Scale bar = 100 μm.** (L)** Quantification of cardiomyocytes' cross-sectional surface area (CSA). Significance was evaluated via the Student's *t* test (F, H, and I) and one-way ANOVA with the post-hoc Bonferroni test (A, C, D, J, and L). *P < 0.05 and **P < 0.01.

To investigate the therapeutic potential of RMST silencing in response to MI, we performed an in vivo loss-of-function study in the murine MI model. AAV6 vectors carrying scrambled control shRNA (MI+RMST NC group) or RMST-specific shRNA (MI+RMST Kd group) were injected into the infarct and the peri-infarct zones of the MI hearts. The TTC staining results showed that there was no statistical difference in the infarct size between the MI+RMST NC group and the MI+RMST Kd group on day 3 after MI (**Figure S5**). The qRT-PCR results showed that the RMST-specific shRNA did not affect the expression of RMST in the BZ of the infarcted hearts on day 3, but notably downregulated it on days 14 and 28 after MI (**Figure 3A**). The echocardiographic assessment showed that in vivo RMST knockdown significantly improved cardiac function, as evidenced by restoring the LVEF and LVFS 14 and 28 days after MI (**Figure 3B-D**). In line with this, Masson staining showed that RMST knockdown significantly reduced the fibrosis size 28 days after MI compared with the RMST NC treatment (**Figure 3E-F**). The survival curve also showed that RMST knockdown reduced the mouse mortality rate after MI compared with the RMST NC treatment (**Figure S6**). Next, immunostaining analysis showed that RMST knockdown markedly inhibited the course of FMT in response to MI on day 28 after MI, as evidenced by a decreased number of α-SMA-positive CFs in the scar regions (**Figure 3G-H**). The mRNA expression of fibrotic genes, including Col1a1, Col3a1, fibronectin 1, and α-SMA, was significantly decreased in RMST knockdown hearts 28 days after MI compared with RMST NC ones (**Figure 3I**). In addition, apparent adverse LV hypertrophy was progressively reversed after RMST knockdown 28 days after MI, as evidenced by a reduction in the ratio of heart weight to body weight (**Figure 3J**) and cardiomyocyte size detected by WGA staining (**Figure 3K-L**). Taken together, these results demonstrate that RMST knockdown improves heart contractile function and reverses the harmful LV remodeling after MI.

### RMST acts as a competing endogenous RNA (ceRNA) for miR-24-3p in CFs

**Figure 4:**
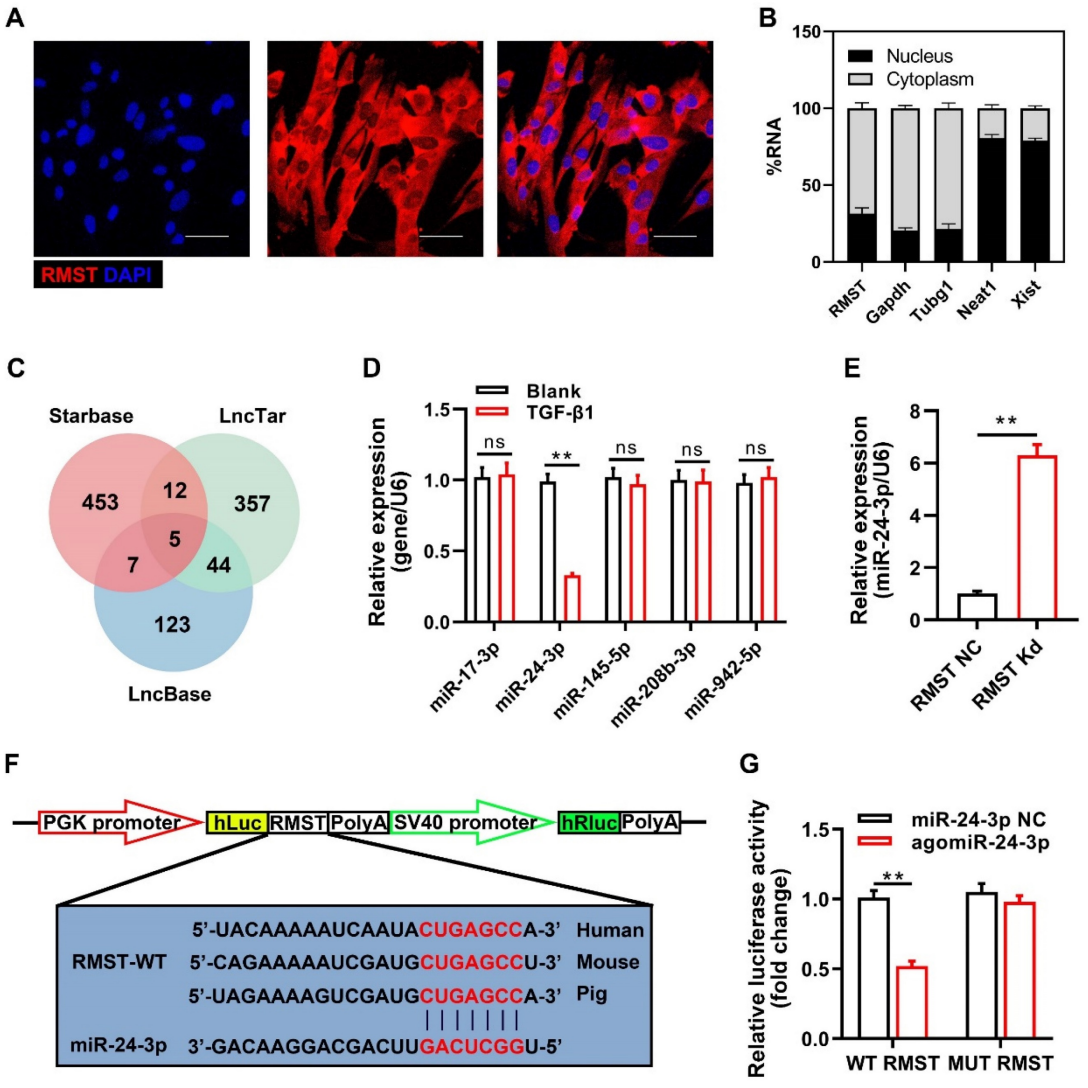
** RMST acts as a ceRNA for miR-24-3p in CFs**. **(A)** The subcellular localization of RMST in CFs was detected by fluorescence in situ hybridization (FISH). Scale bar = 50 μm. **(B)** Percentage of nuclear and cytoplasmic RNA concentrations of RMST, GAPDH, Tubg1 (cytoplasmic marker), Neat1, and Xist (nuclear marker) measured by qRT-PCR. **(C)** Venn diagram depicting RMST-related miRNA candidates derived from bioinformatics analysis.** (D)** The expression of miR-17-3p, miR-24-3p, miR-145-5p, miR-208b-3p, and miR-942-5p in CFs after TGF-β1 treatment was detected by qRT-PCR. **(E)** The miR-24-3p expression in TGF-β1-treated CFs was detected by qRT-PCR after RMST silencing. **(F)** The predicted binding sites of RMST and miR-24-3p. **(G)** Luciferase reporter activities of chimeric vectors carrying the luciferase gene and a fragment of RMST containing wild-type or mutated miR-24-3p binding sites. *n* = 4 independent experiments. Significance was evaluated via Student's *t* test. **P < 0.01. ceRNA, competing endogenous RNAs; Tubg1, tubulin gamma 1; Neat1, nuclear paraspeckle assembly transcript 1; and Xist, X inactive specific transcript.

To identify the specific molecular mechanism of RMST in CF fibrogenesis progress, we detected the subcellular location of RMST by FISH and qRT-PCR. RMST was localized mainly in the cytoplasm in CFs (**Figure 4A-B**). Emerging evidence has demonstrated that cytoplasmic lncRNAs are involved in multiple human diseases, including MI, by competitively binding to miRNAs 17. Therefore, we performed bioinformatics analysis using StarBase, LncTar, and LncBase to identify the candidate miRNAs containing putative binding sites to RMST. Five candidate miRNAs (miR-17-3p, miR-24-3p, miR-145-5p, miR-208b-3p, and miR-942-5p) were found in the coincident part of the bioinformatics analysis Venn diagram (**Figure 4C**). qRT-PCR results showed that during TGF-β1-induced fibrogenesis, the expression of RMST increased, while only the expression of miR-24-3p was downregulated (**Figure 4D**). In addition, RMST knockdown in activated CFs increased the expression of miR-24-3p (**Figure 4E**), while overexpression of RMST using the RMST gene-carrying pcDNA3.1 plasmid decreased the expression of miR-24-3p in activated CFs (**Figure S7A-B**) and enhanced cell proliferation and FMT (**Figure S7C-D**). Therefore, miR-24-3p was selected for further studies. To explore whether RMST acts as a ceRNA for miR-24-3p, we first verified the conservativeness of RMST and miR-24-3p in humans, mice, and pigs (**Figure S8A-B**). Next, a DLR assay was performed, which confirmed that miR-24-3p was a direct downstream target of RMST, as evidenced by the significant decrease in relative luciferase activity in RMST-WT-Luc and agomiR-24-3p co-transfected groups (**Figure 4F-G**). Overall, these findings demonstrate that RMST can directly regulate miR-24-3p as a ceRNA.

### miR-24-3p inhibition abolishes and agomiR-24-3p reproduces the RMST knockdown-mediated effects on TGF-β1-induced CF fibrogenesis by regulating LOX

**Figure 5:**
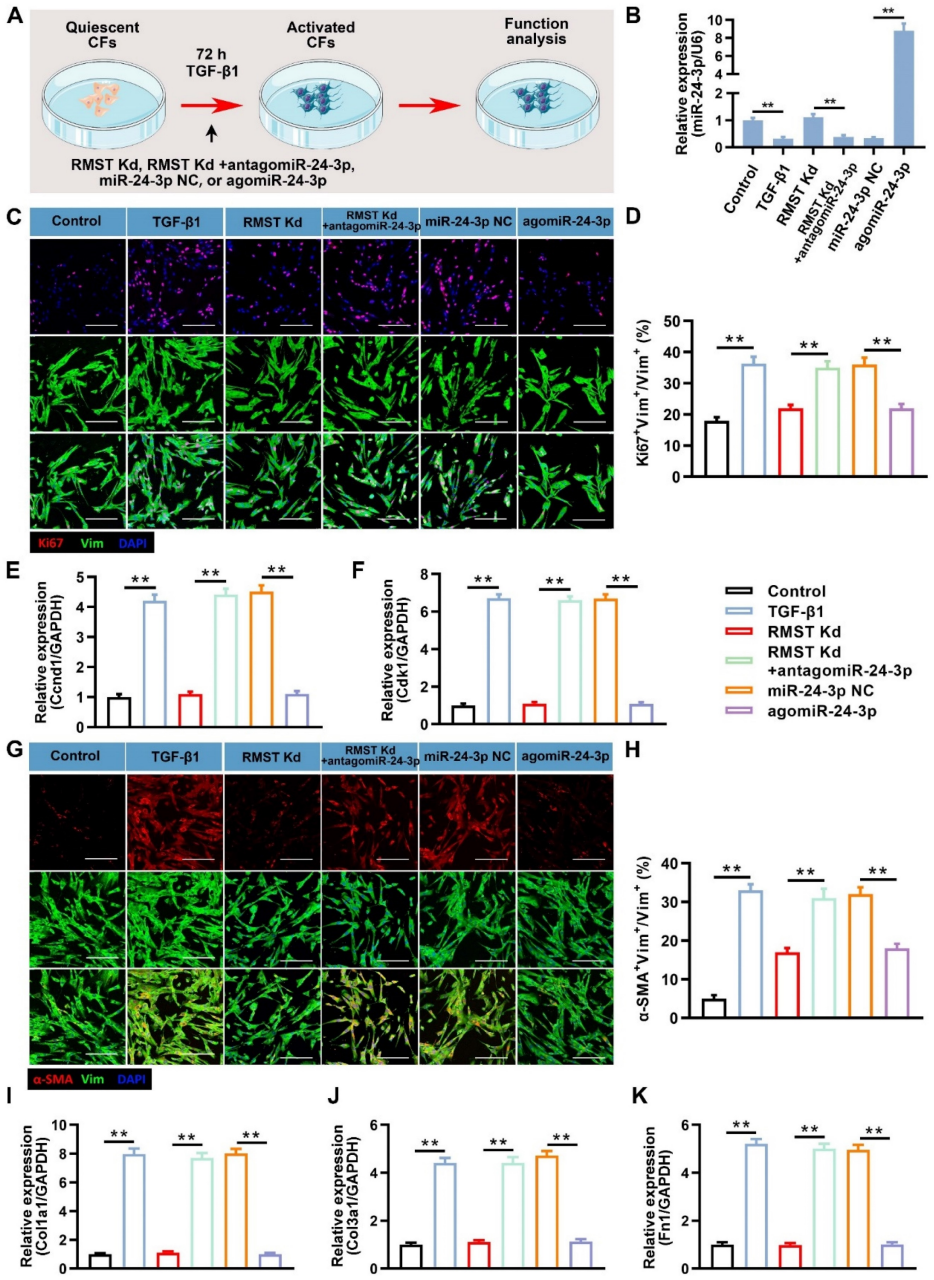
** miR-24-3p loss abolishes and agomiR-24-3p reproduces the RMST knockdown-mediated effects on CF fibrogenesis***.* **(A)** TGF-β1-treated CFs were transfected with miR-24-3p NC (10 nM), antagomiR-24-3p (20 nM), or agomiR-24-3p (10 nM) with or without the knockdown of RMST. CF without any treatment served as a control. **(B)** Quantification of miR-24-3p expression by qRT-PCR under the different treatments. **(C)** CFs were immunofluorescently stained for vimentin and Ki67, and nuclei were counterstained with DAPI. Scale bar = 100 μm.** (D)** Quantification of Ki67-positive CFs. **(E-F)** The mRNA expression of **(E)** Ccnd1 and **(F)** Cdk1 in CFs was detected by qRT-PCR.** (G)** CFs were immunofluorescently stained for α-SMA and vimentin, and nuclei were counterstained with DAPI.** (H)** Quantification of α-SMA-positive cells in CFs. Scale bar = 100 μm.** (I-K)** The mRNA expression of **(I)** Col1a1**, (J)** Col3a1**,** and **(K)** fibronectin in CFs was detected by qRT-PCR**.** *n* = 4 to 5 independent experiments. Significance was evaluated via one-way ANOVA with the post-hoc Bonferroni test. **P < 0.01.

We performed a series of loss- or gain-of-function experiments to investigate the role and specific mechanism of miR-24-3p in RMST knockdown-mediated effects on TGF-β1-induced CF fibrogenesis (**Figure 5A**). The qRT-PCR results showed that antagomiR-24-3p treatment neutralized the upregulation of miR-24-3p expression by RMST knockdown in activated CFs, while agomiR-24-3p transfection dramatically upregulated the miR-24-3p expression (**Figure 5B**). Furthermore, the decreased cell proliferation in activated CFs in the RMST Kd group, which was determined by Ki67 fluorescent immunostaining (**Figure 5C-D**) and mRNA expression of Ccnd1 (**Figure 5E**) and Cdk1 (**Figure 5F**), was abolished in the RMST Kd + antagomiR-24-3p group and reproduced in the agomiR-24-3p group. Congruously, RMST knockdown-mitigated FMT, as determined by α-SMA fluorescent immunostaining (**Figure 5G-H**), wound closure rate assay (**Figure S9A-B**), and fibrin scaffold contraction assay (**Figure S9C-D**), was abrogated with antagomiR-24-3p treatment and reproduced with agomiR-24-3p treatment, which was in line with the change in the mRNA expression of fibrotic genes (Col1a1, Col3a1, and fibronectin 1) (**Figure 5I-K**).

**Figure 6:**
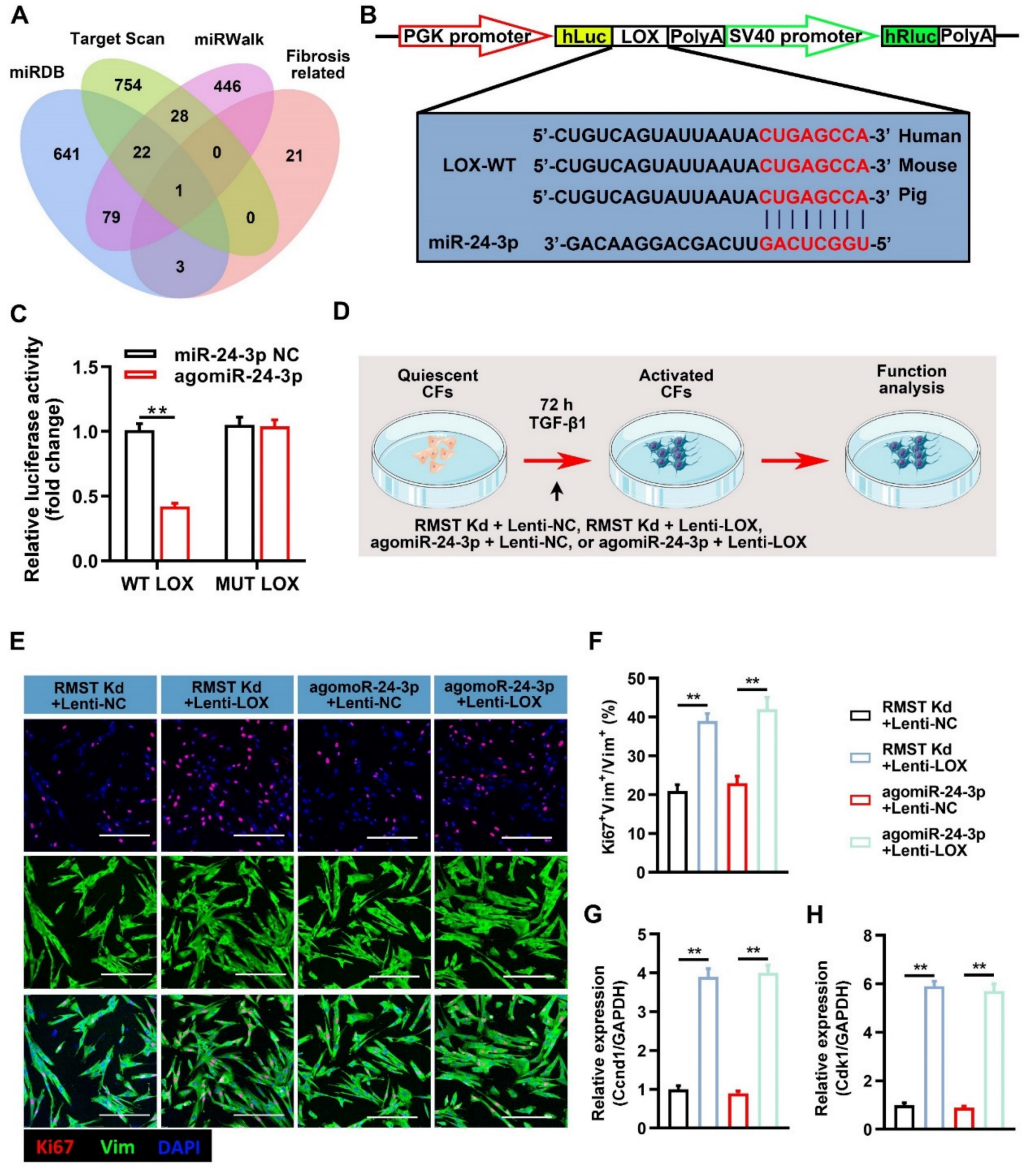
** Lysyl oxidase (LOX) overexpression abolishes the effects of RMST knockdown or agomiR-24-3p treatment on CF fibrogenesis. (A)** Venn diagram depicting miR-24-3p-related mRNA candidates derived from bioinformatics analysis. **(B)** The predicted binding sites of miR-24-3p and LOX. **(C)** Quantification of luciferase activity. **(D)** Workflow of Lenti-LOX treatment. **(E-F)** TGF-β1-treated CFs were immunofluorescently stained for vimentin and Ki67; then, Ki67-positive CFs were quantified. **(G-H)** The mRNA expression of **(G)** Ccnd1 and **(H)** Cdk1 in activated CFs was detected by qRT-PCR. *n* = 4 to 5 independent experiments. Significance was evaluated via one-way ANOVA with the post-hoc Bonferroni test. **P < 0.01.

We next explored the downstream regulatory pathways of miR-24-3p in CF fibrogenesis. A bioinformatics analysis was performed using target prediction databases (including miRBD, Targetscan, and miRWalk) and known fibrosis-related genes 32-37, and we selected LOX as a candidate (**Figure 6A**). LOX is an extracellular copper-dependent enzyme that regulates the crosslinking of structural ECM components in solid organs. The relative luciferase activity was significantly decreased in LOX-WT-Luc and agomiR-24-3p co-transfected groups (**Figure 6B-C**), indicating the existence of a site on the LOX sequence that can directly bind to miR-24-3p. RMST knockdown decreased the mRNA and protein expression of LOX in the activated CFs, while RMST overexpression increased it (**Figure S10A-C**). Next, we performed a series of gain-of-function experiments to verify the effect of LOX (**Figure 6D**), and LOX was overexpressed using a lentiviral system. Measurements of Ki67-positive CFs (**Figure 6E-F**), mRNA expression of Ccnd1 (**Figure 6G**) and Cdk1 (**Figure 6H**), α-SMA-positive CFs (**Figure S11A-B**), fibrin scaffold contraction (**Figure S11C-D**), wound healing assay (**Figure S11E**), mRNA expression of fibrotic genes (Col1a1, Col3a1, fibronectin 1, and α-SMA) (**Figure S12A-D**), and protein expression of LOX, MMP-2, and MMP-9 (**Figure S12E-H**) in the RMST Kd+Lenti-NC group or agomiR-24-3p+Lenti-NC group were reversed in the RMST Kd+Lenti-LOX group or agomiR-24-3p+ Lenti-LOX group. Taken together, these results demonstrate that RMST knockdown relieves CF fibrogenesis through the RMST/miR-24-3p/LOX signaling pathway.

### Therapeutic silencing RMST alleviates cardiac fibrosis and improves cardiac function in a female porcine MI model

**Figure 7:**
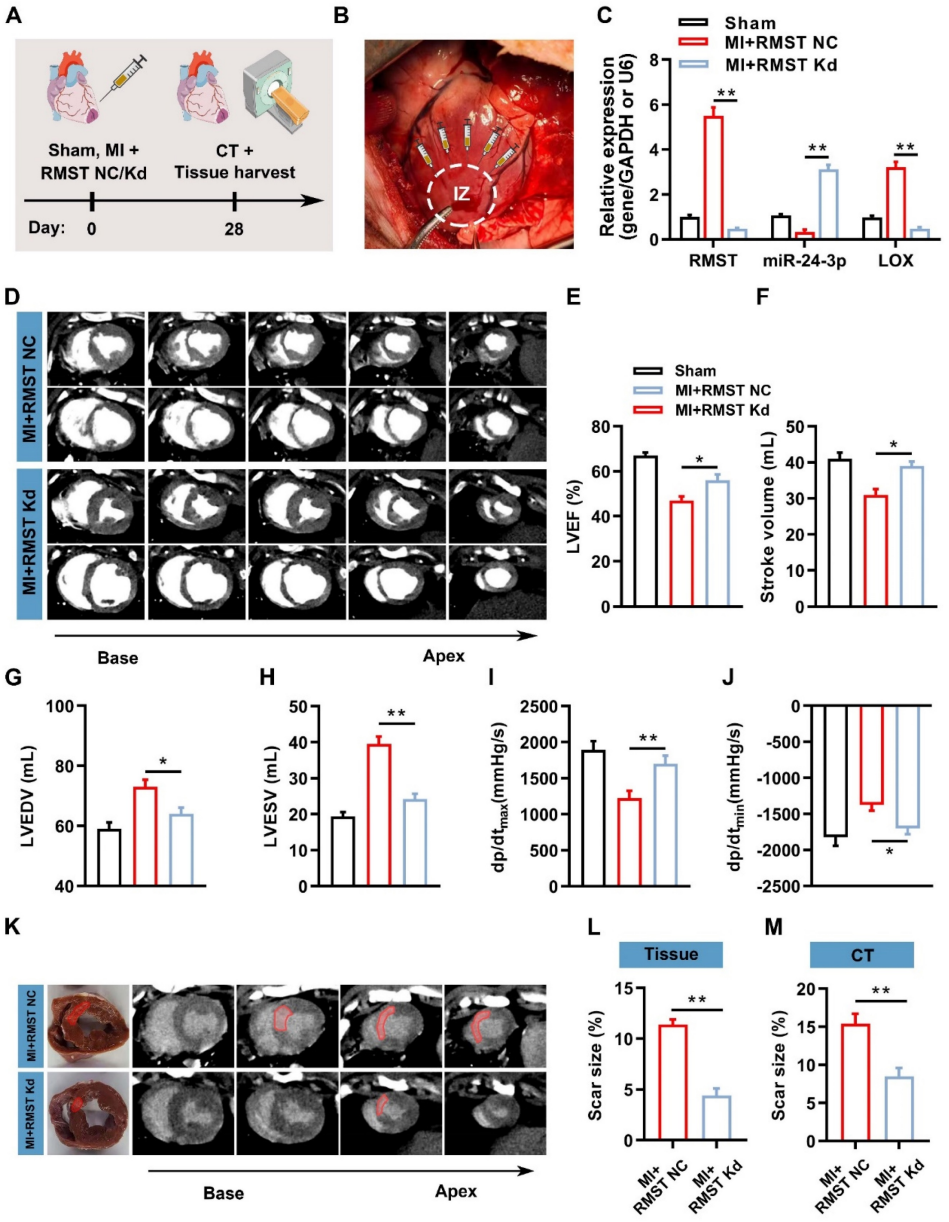
** RMST knockdown mitigates cardiac infarct size and ameliorates myocardial function in a porcine MI model**. **(A)** Schematic representation of the pig study. **(B)** After MI surgery, pig hearts were administered with 1 mL of AAV6 vectors carrying scrambled shRNA (MI+RMST NC group) or an equal amount of AAV6 vectors carrying RMST-specific shRNA (MI+RMST Kd group) by intramyocardial injection. Sham control (sham group) was also applied. **(C)** The mRNA expression of RMST, miR-24-3p, and LOX was detected by qRT-PCR on day 28 after MI. RMST and LOX expression was normalized to GAPDH, and miR-24-3p expression was normalized to U6. **(D)** Cardiac short-axis high-resolution computed tomography (CT) images of LV end-diastole and end-systole on day 28 after MI. **(E-H)** Quantification of **(E)** LVEF, **(F)** stroke volume,** (G)** LV end-diastolic volume (LVEDV), and **(H)** LV end-systolic volume (LVESV) assessed by CT. **(I)** The maximum rising rate of LV pressure (+dP/dt_max_) and **(J)** maximum declining rate of LV pressure (dP/dt_min_) were recorded and calculated on day 28 after MI.** (K)** Representative images of fresh cardiac tissues and delayed enhancement cardiac CT of scar size analysis on day 28 after MI.** (L-M)** Quantification of scar size. *n* = 6 animals per group. Significance was evaluated via Student's *t* test (L and M) and one-way ANOVA with the post-hoc Bonferroni test (C, E-J). * *P* < 0.05; ** *P* < 0.01. IZ, infarct zone.

To investigate whether RMST silencing can exert therapeutic effects to alleviate cardiac fibrosis and improve contractile function in the preclinical large animal model, AAV6 vectors carrying scrambled control shRNA (MI+RMST NC group) or RMST-specific shRNA (MI+RMST Kd group) were injected into the infarct and the peri-infarct zones of the MI pig hearts (**Figure 7A-B**). qRT-PCR results showed that the RMST-specific shRNA downregulated the mRNA expression of RMST and LOX and upregulated the expression of miR-24-3p in the BZ of the infarcted pig hearts on day 28 after MI compared with the RMST NC treatment (**Figure 7C**). Cardiac function was evaluated 28 days after MI via CT and hemodynamic measurements. Compared with the RMST NC treatment, RMST knockdown significantly enhanced LVEF and stroke volume, reduced LV end-diastolic volume (LVEDV) and LV end-systolic volume (LVESV), and conserved the maximum rising rate of LV pressure (dP/dt_max_) and maximum declining rate of LV pressure (dP/dt_min_) (**Figure 7D-J**). Similarly, the evaluation of myocardial perfusion and wall motion detected by CT analysis showed that these values on day 28 after MI were all enhanced in the RMST knockdown pigs compared with the RMST NC animals (**Figure S13A-C**). The analyses of fresh heart slides and delayed enhancement cardiac CT images (**Figure 7K**) showed that RMST knockdown significantly reduced pig cardiac scar size after MI (**Figure 7L-M**).

**Figure 8:**
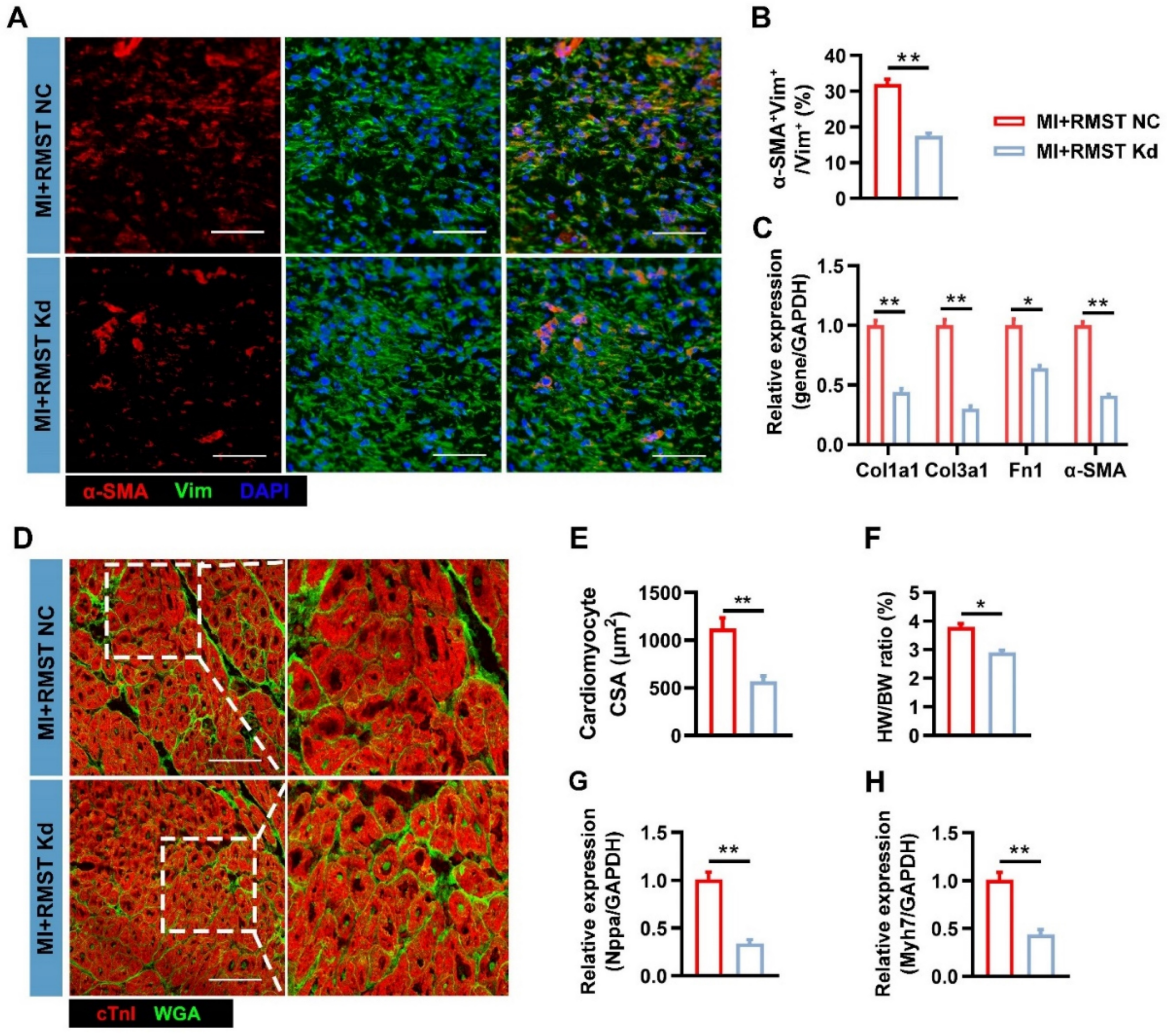
**RMST knockdown inhibits CF FMT and cardiomyocyte hypertrophy in porcine hearts after MI**. **(A)** Sections from the BZ of infarcted porcine hearts were immunofluorescently stained for α-SMA and vimentin on day 28 after MI. Scale bar = 200 μm.** (B)** Quantification of α-SMA-positive CFs. **(C)** The mRNA expression of Col1a1**,** Col3a1**,** fibronectin 1, and α-SMA in the BZ of infarcted porcine hearts was detected by qRT-PCR**. (D)** Sections from the BZ (28 days after MI) were stained with WGA to identify the cellular borders and with cardiac troponin I (cTnI) to visualize CMs. Scale bar = 200 μm. **(E)** Cardiomyocytes' cross-sectional surface area (CSA) was measured. **(F)** The HW/BW ratio was measured on day 28 after MI.** (G-H)** Quantification of mRNA expression of **(G)** hypertrophy-related genes natriuretic peptide precursor A (Nppa) and **(H)** β myosin heavy chain 7 (Myh7) by qRT-PCR. *n* = 6 per experimental group. Significance was evaluated via Student's *t* test. *P < 0.05; **P < 0.01.

Moreover, RMST knockdown reduced the number of α-SMA-positive CFs in the BZ on day 28 after MI (**Figure 8A-B**) compared with the RMST NC group, indicating that RMST silencing impeded the CF FMT progress. Consistently, the mRNA expression of fibrotic genes, including Col1a1, Col3a1, fibronectin 1, and α-SMA, was downregulated in the BZ of the infarcted pig hearts after RMST knockdown compared with RMST NC treatment (**Figure 8C**). The WGA staining assessment showed that RMST knockdown reduced the cross-sectional surface areas of cardiomyocytes in the BZ of the infarcted hearts (**Figure 8D-E**). In line with this, HW/BW ratio indicated that RMST silencing was able to reverse cardiac hypertrophy, which was also confirmed by a decrease in mRNA expression of hypertrophy-related genes, including natriuretic peptide precursor A (Nppa) and β myosin heavy chain 7 (Myh7) (**Figure 8F-H**). Collectively, these results demonstrate that RMST knockdown improves heart contractile function and reverses the harmful LV remodeling in pigs after MI, exhibiting promising therapeutic potential against MI.

## Discussion

In the current study, we first reported on the characterization of a crucial cardiac fibrosis regulator named lncRNA RMST and investigated the underlying mechanisms. The main findings are as follows: (i) RMST expression was dramatically increased and associated with cardiac fibrosis in mice and pigs after MI. (ii) In vitro RMST knockdown reduced TGF-β1-induced CF fibrogenesis, including the reduction in cell proliferation, FMT, and ECM production. (iii) RMST knockdown decreased fibrosis size and improved cardiac performance in MI mice. (iv) Bioinformatics and gain- or loss-of-function analysis identified RMST as a ceRNA of miR-24-3p, which could regulate cardiac fibrosis through the LOX signaling pathway. (v) RMST knockdown prevented cardiac fibrosis to a certain extent and improved cardiac function in MI pigs. These findings provide evidence that RMST silencing may be a promising strategy for MI therapy.

Accumulating evidence demonstrates that lncRNAs are involved in multiple human diseases and disorders, such as cancer, metabolic disorders, and cardiovascular diseases 14, 24, 38. RMST, one kind of lncRNA, was first identified during dopaminergic neuron development 38. Here, we further linked the inhibition of RMST to cardiac fibrosis reduction and heart function recovery after MI. We provided corroborating evidence that RMST silencing reduced fibrosis through regulating CF, such as reducing the expression of proliferation marker Ki67 and FMT indicator α-SMA, cell migration and contractile capacity, and production of Col1a1, Col3a1, and fibronectin 1 in CFs, which were induced by TGF-β1 treatment in vitro.

Conversely, RMST overexpression further aggravated fibrosis in TGF-β1-treated CFs. Furthermore, we verified the therapeutic function of RMST silencing on MI in a mouse model. Echocardiographic results showed that RMST knockdown conserved mouse cardiac function after MI; Masson and immunofluorescence staining assessment confirmed that RMST knockdown dramatically decreased cardiac fibrosis; and the production of ECM detected by qRT-PCR was also reduced by RMST knockdown. In addition, the evaluation of the clinical translation potential of RMST silencing in a pig MI model also revealed that RMST knockdown reduced cardiac fibrosis through modulating FMT and ameliorated heart function. These results confirmed our hypothesis that RMST silencing could regulate CF activation to improve cardiac fibrosis and contractile function after MI.

To the best of our knowledge, this is the first study focusing on the lncRNA's therapeutic effect in a preclinical pig MI model. Experimental data obtained from small animal models are challenging to achieve clinical translation because of the enormous structural, electrophysiological, and hemodynamic differences between rodents and humans 19. Therefore, our data gained from the large animal (pig) model, which has higher clinical relevance to human diseases than the small animal model, indicate the clinical application potential of RMST silencing for the treatment of MI. Here, we used female pigs as experimental animals, according to previous reports 29, 39, 40. This was done mainly because male pigs emit unpleasant odors and are aggressive during rearing and experimental procedures 41. Although one recent study showed that sex (female, male, castrated male) had no impact on infarct size, coronary microvascular damage, and cardioprotection through ischemic preconditioning in pigs undergoing MI 42, we did not exclude the influence of sex differences on the therapeutic effects of RMST silencing against MI injury, which is a limitation of this study. Previous studies have reported that lncRNAs, such as Xist, MALAT1, and HULC, lack conservation between different species 43-45; however, it is worth noting that many lncRNAs still contain strongly conserved elements and exhibit similar biological functions 46, 47. Interestingly, in this study, we found that although the entire length of RMST is poorly conserved, the sequence of the binding site of miR-24-3p is conserved across species, including mice, pigs, and humans. This illustrates that RMST may have a similar biological function among humans, mice, and pigs, which is also supported by the evidence that the effect of RMST knockdown on reducing cardiac fibrosis and restoring cardiac function in MI pigs is consistent with that in MI mice.

There is cumulative evidence showing that lncRNAs could act as ceRNAs in human diseases, including fibrosis 17, 48, 49. Here, we identified RMST as a ceRNA to regulate the LOX pathway by sponging miR-24-3p in cardiac fibrosis after MI. Through the bioinformatics analysis and DLR assay, we first confirmed the direct binding sites between RMST and miR-24-3p; loss- or gain-of-function experiments further showed that miR-24-3p inhibition by antagomiR-24-3p treatment abolished, while agomiR-24-3p reproduced, the RMST knockdown-mediated effects on TGF-β1-induced CF fibrosis. In the pig MI model, we demonstrated that RMST knockdown significantly upregulated the expression of miR-24-3p in the BZ of the infarcted pig hearts, which was consistent with the in vitro finding that miR-24-3p was a ceRNA of RMST. A previous study also supported these findings that miR-24-3p restored cardiac function after MI by alleviating cardiomyocyte apoptosis and cardiac fibrosis 50.

LOX is a copper-dependent amine oxidase whose primary function is the covalent crosslinking of collagen in the ECM 51. Many studies have confirmed that the LOX family promotes parenchymal organ fibrosis 52-54. In cardiac fibrosis, LOX inhibition has been shown to suppress fibrosis progression and accelerate its reversal in a rodent model 55. Consistently, in our study, LOX was identified as the downstream target gene of miR-24-3p by bioinformatics analysis, which was further confirmed by DLR assay. A series of gain-of-function experiments also verified that LOX overexpression abolished the effects of RMST knockdown and agomiR-24-3 treatment on inhibiting CF fibrosis. Furthermore, our results revealed that RMST knockdown significantly downregulated the expression of LOX in the BZ of the infarcted pig hearts. These findings indicate that RMST regulates cardiac fibrosis through the RMST/miR-24-3p/LOX signaling pathway.

After excessive deposition of ECM during MI, scar tissue without contractility destroys the coordination of ventricular motion, and fibroblasts/myofibroblasts within the scar tissue release maladaptive proinflammatory and prohypertrophic signals, increasing the ventricular load and promoting further apoptosis and the hypertrophy of cardiomyocytes 56-58. Cardiomyocyte hypertrophy further reduces ventricular systolic function and decreases cardiac output. The current study found that RMST knockdown prevented cardiomyocyte hypertrophy, as evidenced by the reduction in cardiomyocytes' cross-sectional surface area and heart weight/body weight ratio. Considering the interaction between fibroblasts and cardiomyocytes 57, 59, reversed cardiac hypertrophy mediated by RMST knockdown may be achieved by reducing fibrosis during cardiac remodeling, which subsequently results in improved heart performance. In addition, we found that RMST expression was slightly increased in cardiomyocytes after MI. Due to considerations of length, whether RMST knockdown directly affects cardiomyocyte apoptosis and hypertrophy was not explored in this research, but will be investigated in a future study.

It is well established that AAV is less immunogenic than adenovirus and has been used clinically 60, 61. The expression of the target gene carried by the AAV-mediated delivery system takes effect 7-10 days after local injection and is maintained for several months 25, 62, 63. Therefore, no decrease in RMST expression was observed in the heart on day 3 after AAV6-RMST shRNA treatment, which was consistent with the unchanged myocardial infarct size. In addition, fibroblast activation and fibrous scar formation in the early phase of MI are essential, as they maintain cardiac integrity and prevent cardiac rupture due to the massive death of cardiomyocytes 64. Thus, AAV6-RMST shRNA administration immediately after MI may be the best therapeutic window for inhibiting excessive fibrotic responses. However, AAV6-associated gene therapy in hearts still faces some limitations. In the current study, AAV6-RMST shRNA was delivered by in situ intramyocardial injections for RMST silencing in vivo. This delivery strategy is mainly suitable for patients requiring thoracotomy, including coronary artery bypass grafting or heart valve replacement. A safer and highly efficient gene silencing system with cardiac targeting should be developed for clinical application in the future.

In summary, our present work identified lncRNA RMST as a novel regulator of pathological cardiac remodeling in response to MI. RMST exerts its function via sponging miR-24-3p, consequently regulating LOX-mediated CF proliferation, FMT progression, and ECM production. These results illustrate that interfering with RMST expression may offer a promising therapeutic strategy for treating MI.

## Supplementary Material

Supplementary figures and tables.

## Abbreviations

ECM: extracellular matrix
MI: myocardial infarction
lncRNAs: long noncoding RNAs
CF: cardiac fibroblast
FMT: fibroblast-to-myofibroblast transition
RMST: rhabdomyosarcoma 2-associated transcript
LOX: lysyl oxidase
RNA seq: RNA sequencing
FISH: fluorescence in situ hybridization
DLR: dual-luciferase reporter
AAV6: adeno-associated virus serotype 6
LVEF: left ventricular ejection fraction
LVFS: left ventricular fractional shortening
CT: computed tomography
Col1a1: collagen type I alpha 1
Col3a1: collagen type III alpha 1
α-SMA: alpha-smooth muscle actin
PCGs: protein-coding genes
Ccnd1: cyclin D1
Cdk1: cyclin-dependent kinase 1
