# Supplementary material for: Therapeutic silencing of lncRNA RMST alleviates cardiac fibrosis and improves heart function after myocardial infarction in mice and swine
Source: Theranostics. 2023 Jun 26;13(11):3826–43. doi: 10.7150/thno.82543 (PMC10334841; doi:10.7150/thno.82543)
Supplement: Supplementary file 1 — Supplementary figures and tables. [file thnov13p3826s1.pdf]

Figure S1

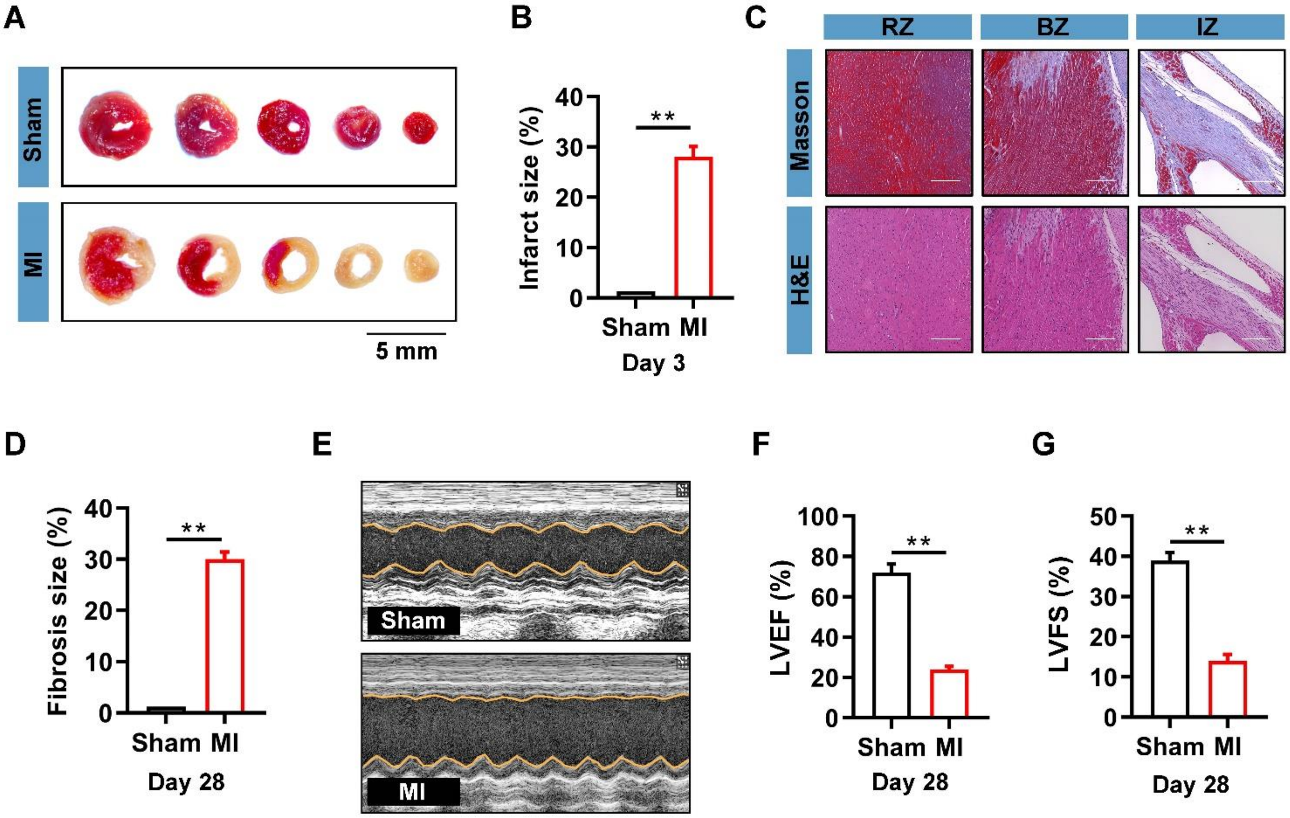

Figure S1. Assessment of infarct size and cardiac function in mouse hearts after myocardial infarction (MI). The mice were divided into the sham and MI groups. **(A)** The infarct size of MI hearts was detected by triphenyl tetrazolium chloride (TTC) staining on day 3 after MI. Scale bar = 5 mm. (**B**) Quantification of infarct size. **(C)** Cardiac fibrosis was detected by Masson and hematoxylin and eosin (H&E) staining on day 28 after MI. Scale bar = 500 μm. **(D)** Quantification of fibrosis size. **(E)** Representative images of echocardiography on day 28 after MI or sham surgery. (**F–G**) Quantification of (**F**) left ventricular (LV) ejection fraction (LVEF) and (**G**) LV fractional shortening (LVFS) measured by echocardiography on day 28 after MI or sham surgery. *n* = 5–8 in each group. Significance was evaluated via the Student's *t* test. **$P < 0.01$. RZ, remote zone; BZ, border zone; and IZ, infarct zone.

Figure S2

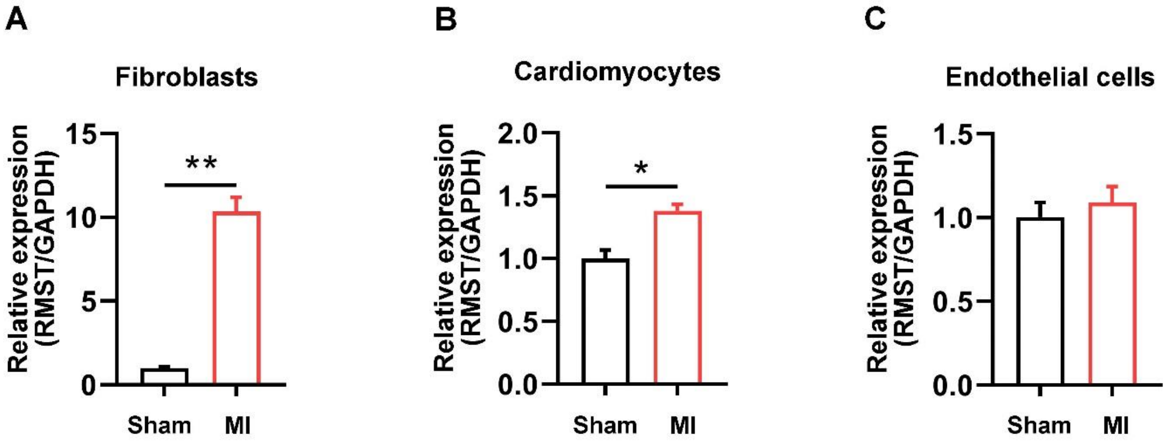

Figure S2. LnRNA RMST expression in different types of mouse cardiac cells. (A–C) The mRNA expression of RMST in **(A)** fibroblasts**, (B)** cardiomyocytes, and **(C)** endothelial cells isolated from mouse hearts was detected by qRT-PCR on day 28 after MI or sham surgery. $n = 4$ in each group. Significance was evaluated via Student's t test. $^{**}P < 0.01$.

Figure S3

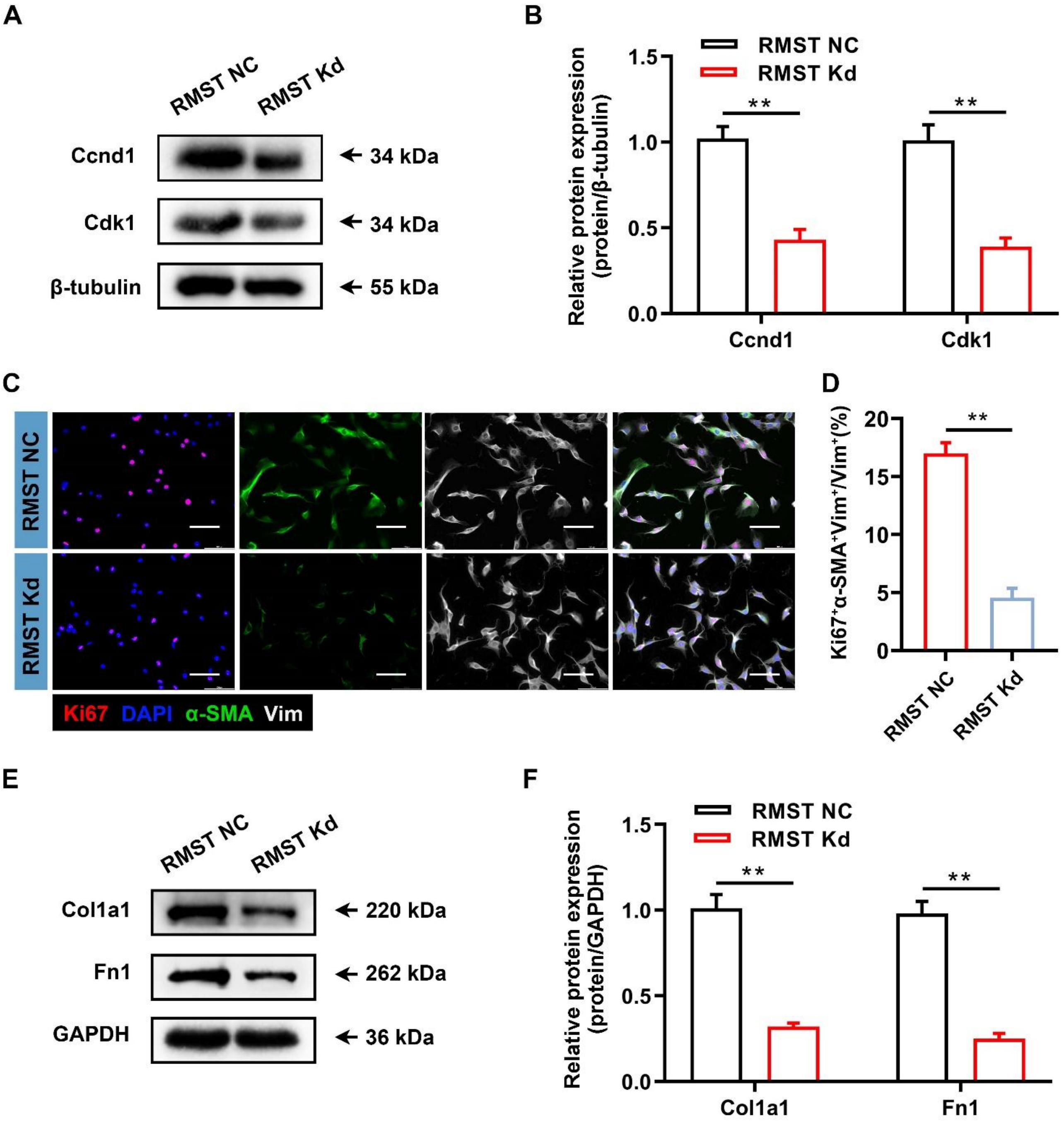

Figure S3. RMST knockdown (Kd) reduces the activation and extracellular deposition in TGF-β1–treated cardiac fibroblasts (CFs). **(A)** The protein expression of cyclin D1 (Ccnd1) and cyclin-dependent kinase 1 (Cdk1) in TGF-β1–treated CFs was detected by western blot. **(B)** Quantification of protein bands. **(C)** TGF-β1–treated CFs were immunofluorescently stained for the expression of vimentin (fibroblast marker), α-SMA (FMT indicator), and Ki67 (cell proliferation marker), and nuclei were counterstained with DAPI. Scale bar = 100 µm. **(D)** Quantification of Ki67^{+}/α-SMA^{+} cells in CFs. **(E)** The protein expression of collagen type I alpha 1 (Col1a1) and fibronectin 1 (Fn1) in TGF-β1–treated CFs was detected by western blot. **(F)** Quantification of protein bands. *n* = 4 independent experiments. Significance was evaluated via Student's *t* test. $^{**}P < 0.01$. NC, negative control; Vim, vimentin.

Figure S4

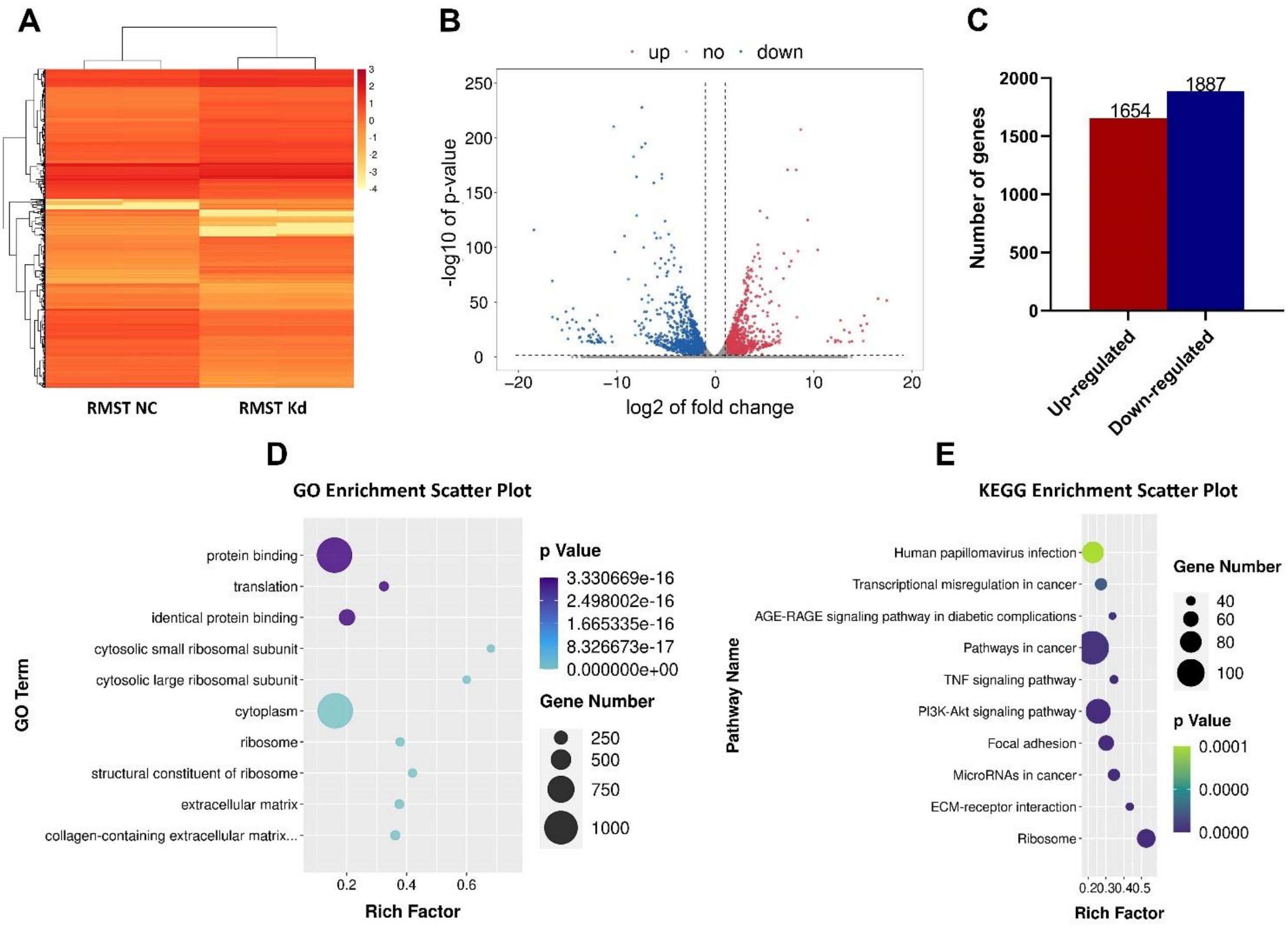

Figure S4. RMST regulates CF gene programs. (A) The heat map and **(B)** the volcano plot show differentially expressed genes after RMST knockdown. **(C)** A total of 1654 upregulated genes and 1887 downregulated genes were detected by gene programs after RMST knockdown. **(D)** GO analysis of the differentially expressed genes is shown in the enrichment scatter plot. **(E)** KEGG pathway analysis of the differentially expressed genes is demonstrated in the enrichment scatter plot. GO, Gene Ontology; KEGG, Kyoto Encyclopedia of Genes and Genomes.

Figure S5

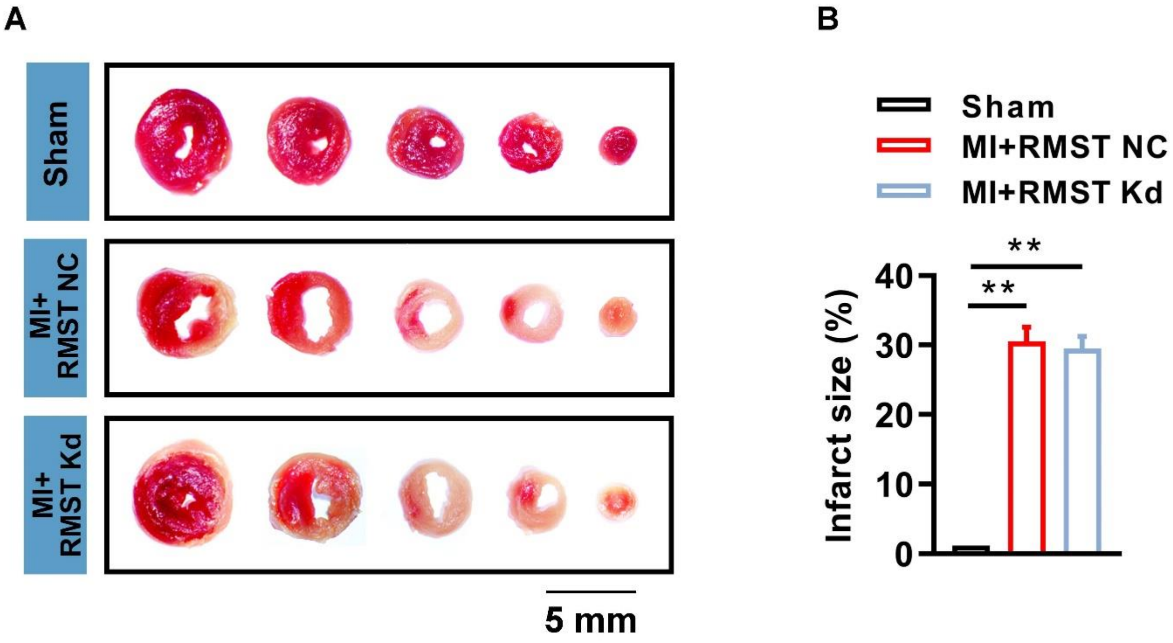

Figure S5. Assessment of infarct size in mouse MI hearts after RMST knockdown. (A) Infarct size was detected by TTC staining on day 3 after MI. Scale bar = 5 mm. **(B)** Quantification of infarct size. *n* = 5 in each group. Significance was evaluated via one-way ANOVA with the post-hoc Bonferroni test. **$P < 0.01$.

Figure S6

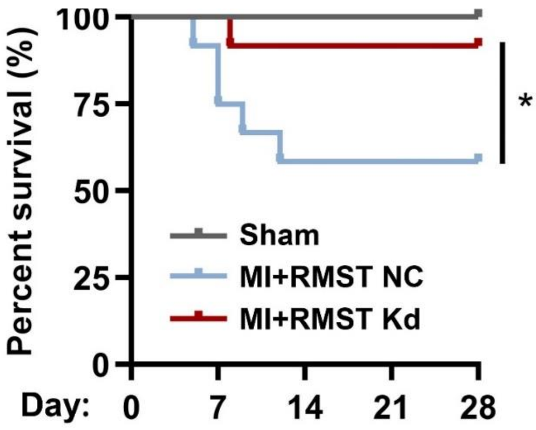

Figure S6. RMST knockdown reduced the mouse mortality rate after MI. The survival curve of mice among the different groups. *n* = 12 in each group. Significance was evaluated via the log-rank test. *$P < 0.05$.

Figure S7

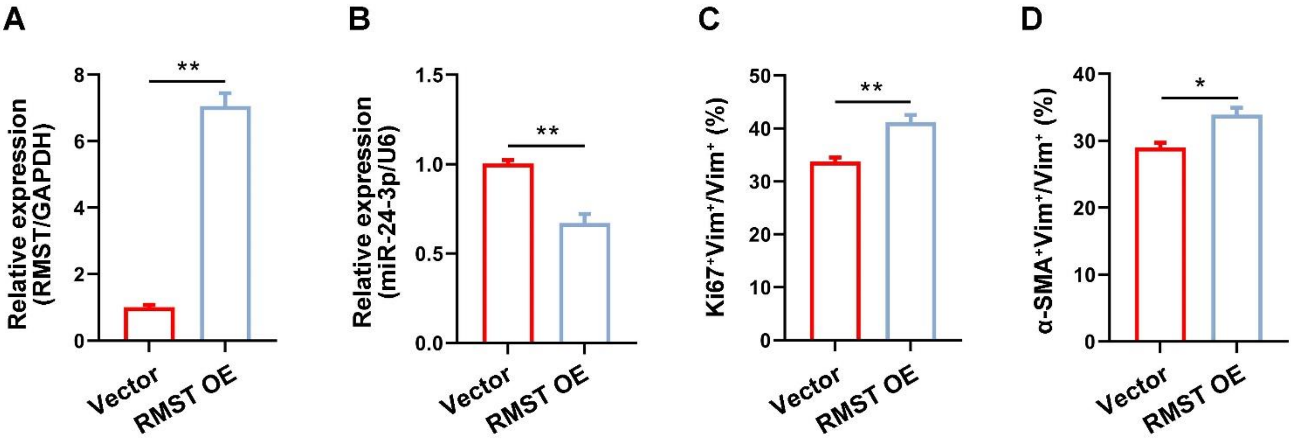

Figure S7. RMST overexpression promoted CF proliferation and fibroblast-to-myofibroblast transition (FMT). CFs were transfected with the pcDNA3.1-RMST plasmid for RMST overexpression (RMST OE) and empty pcDNA3.1 plasmid as a control (vector). **(A)** The mRNA expression of RMST in CFs was detected by qRT-PCR. **(B)** The expression of miR-24-3p in TGF-β1–treated CFs was detected by qRT-PCR. **(C)** TGF-β1–treated CFs were immunofluorescently stained for the expression of vimentin and Ki67, and Ki67-positive cells in CFs were quantified. **(D)** TGF-β1–treated CFs were immunofluorescently stained for the expression of α-SMA and vimentin, and α-SMA–positive cells in CFs were quantified. $n = 4$ independent experiments. Significance was evaluated via Student's *t* test. $^{**}P < 0.01$.

Figure S8

A

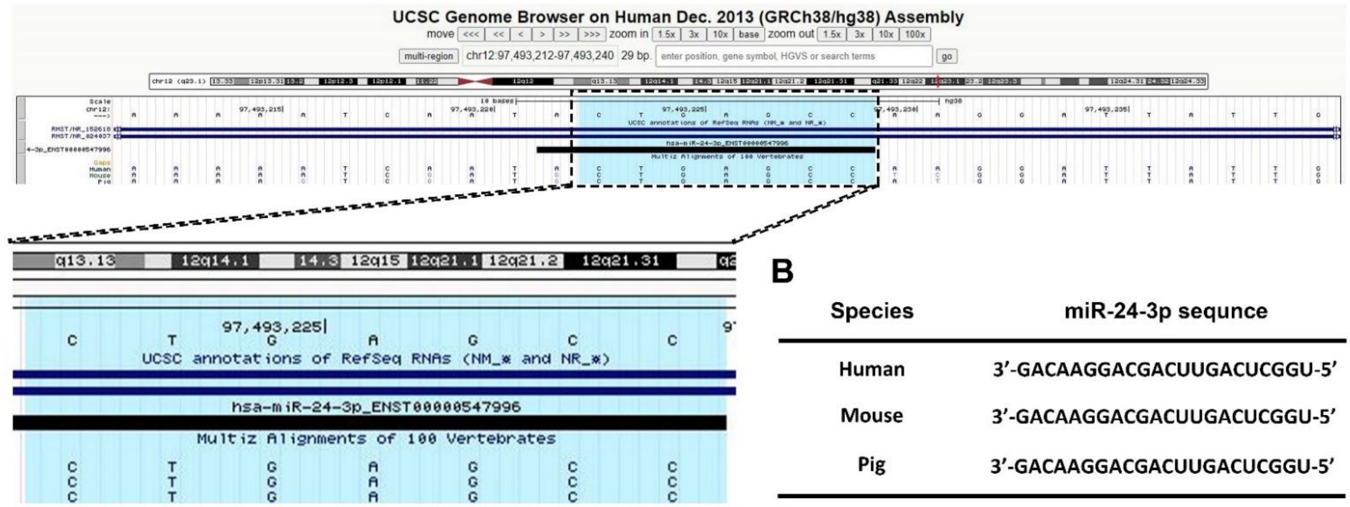

B

Species	miR-24-3p sequnce
Human	3'-GACAAGGACGACUUGACUCGGU-5'
Mouse	3'-GACAAGGACGACUUGACUCGGU-5'
Pig	3'-GACAAGGACGACUUGACUCGGU-5'

Figure S8. Conservation of RMST in the binding site of miR-24-3p. (A) A snapshot of the binding sites between RMST and miR-24-3p from mouse genome (2011 assembly) in UCSC Genome Browser. **(B)** miR-24-3p conservation in humans, mice, and pigs.

Figure S9

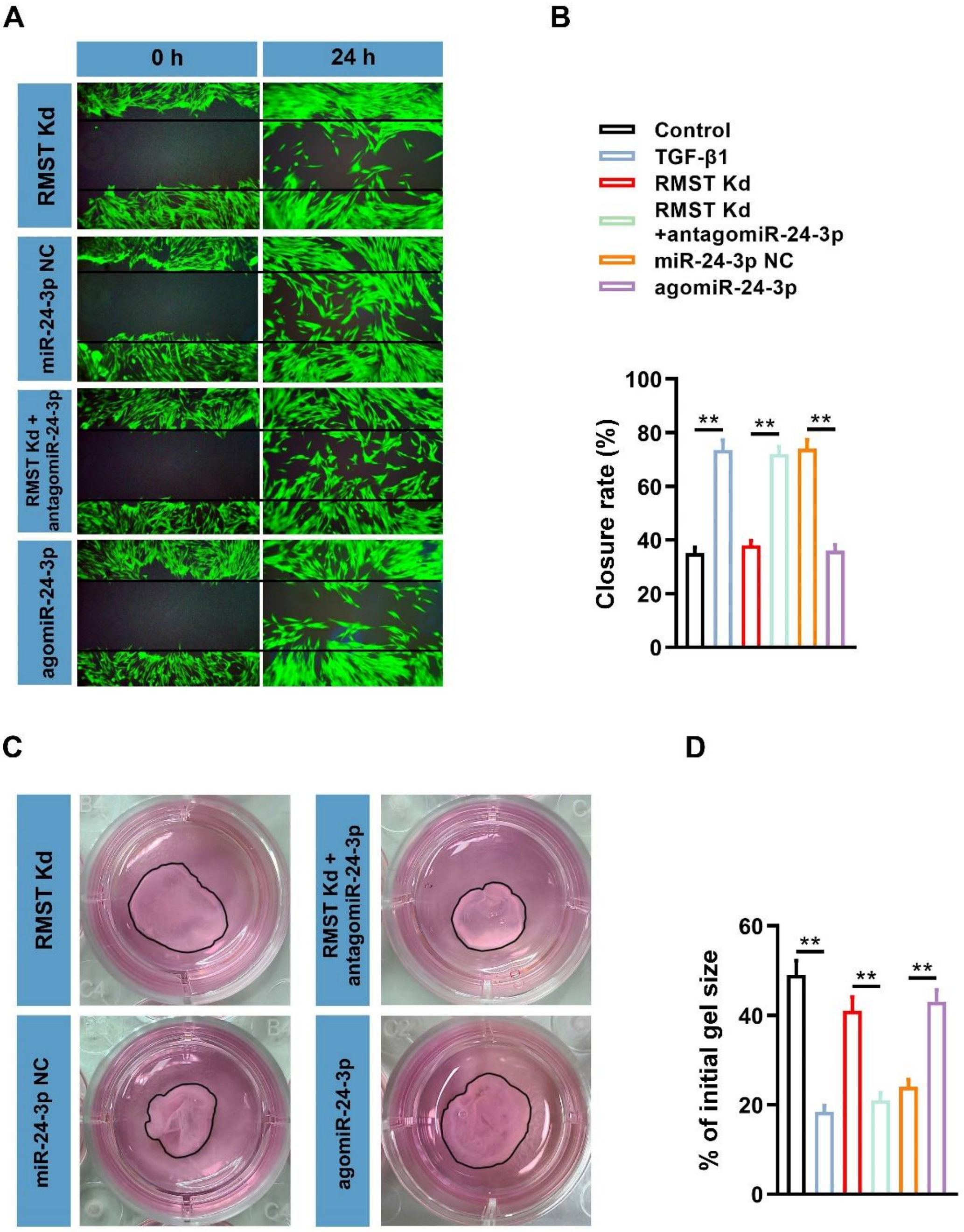

Figure S9. miR-24-3p loss abolishes and agomiR-24-3p reproduces the RMST knockdown–mediated effects on CF migration and contraction. TGF-β1–treated CFs were transfected with miR-24-3p NC, antagomiR-24-3p, or agomiR-24-3p with or without the knockdown of RMST. CFs without any treatment served as a control. **(A)** The migration ability of CFs was assessed by wound healing assay. **(B)** Quantification of closure rate. **(C)** The contractile ability of CFs was assessed by shrink ring assay. **(D)** Quantification of fibrin scaffold contraction. n = 5 independent treatments. Significance was evaluated via one-way ANOVA with the post-hoc Bonferroni test. $^{**}P < 0.01$.

Figure S10

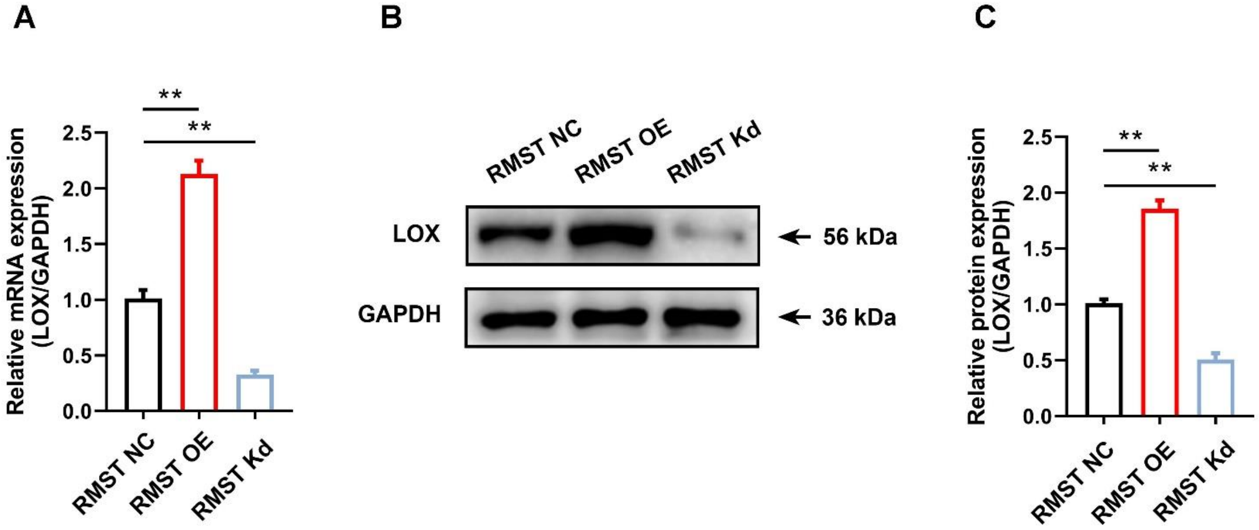

Figure S10. The expression of LOX was correlated with RMST level in CFs. (A) The mRNA expression of lysyl oxidase (LOX) in TGF-β1–treated CFs was detected by qRT-PCR after RMST overexpression (OE) or knockdown (Kd). **(B)** The protein expression of LOX in TGF-β1–treated CFs was detected by western blot after RMST overexpression or knockdown. **(C)** Quantification of protein bands. *n* = 4 independent experiments. Significance was evaluated via one-way ANOVA with the post-hoc Bonferroni test. $^{**}P < 0.01$.

Figure S11

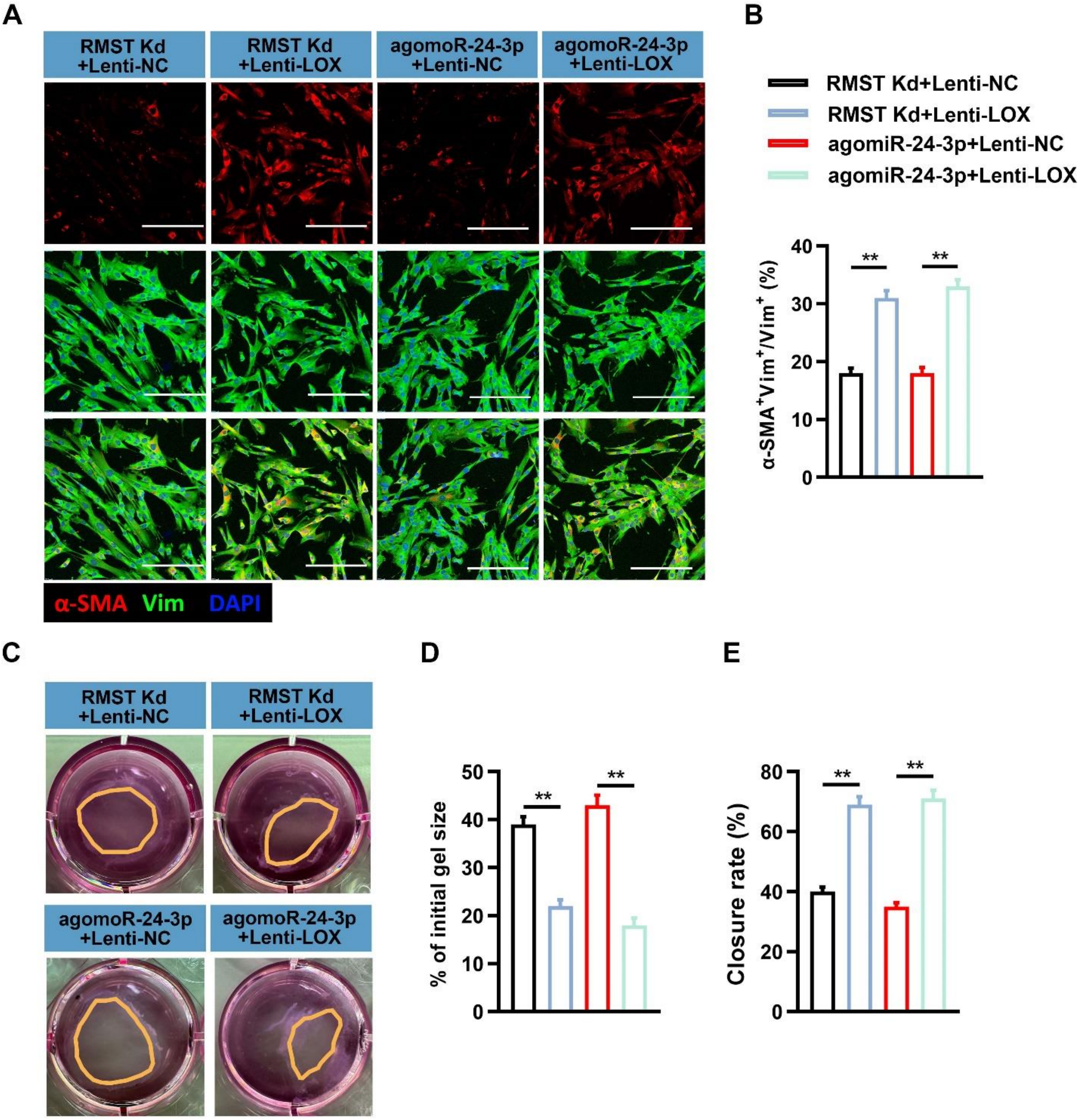

Figure S11. LOX overexpression abolishes the effects of RMST knockdown or agomiR-24-3p treatment on CF fibrogenesis. (A) TGF-β1–treated CFs were immunofluorescently stained for α-SMA and vimentin expression, and nuclei were counterstained with DAPI. Scale bar = 100 μm. **(B)** Quantification of α-SMA^{+} cells in CFs. **(C)** The contractile ability of TGF-β1–activated CFs was assessed by shrink ring assay. (**D**) Quantification of fibrin scaffold contraction. **(E)** The migration ability of TGF-β1–treated CFs was assessed by the wound healing assay, and the closure rate was quantified. *n* = 5 independent treatments. Significance was evaluated via one-way ANOVA with the post-hoc Bonferroni test. $^{**}P < 0.01$.

Figure S12

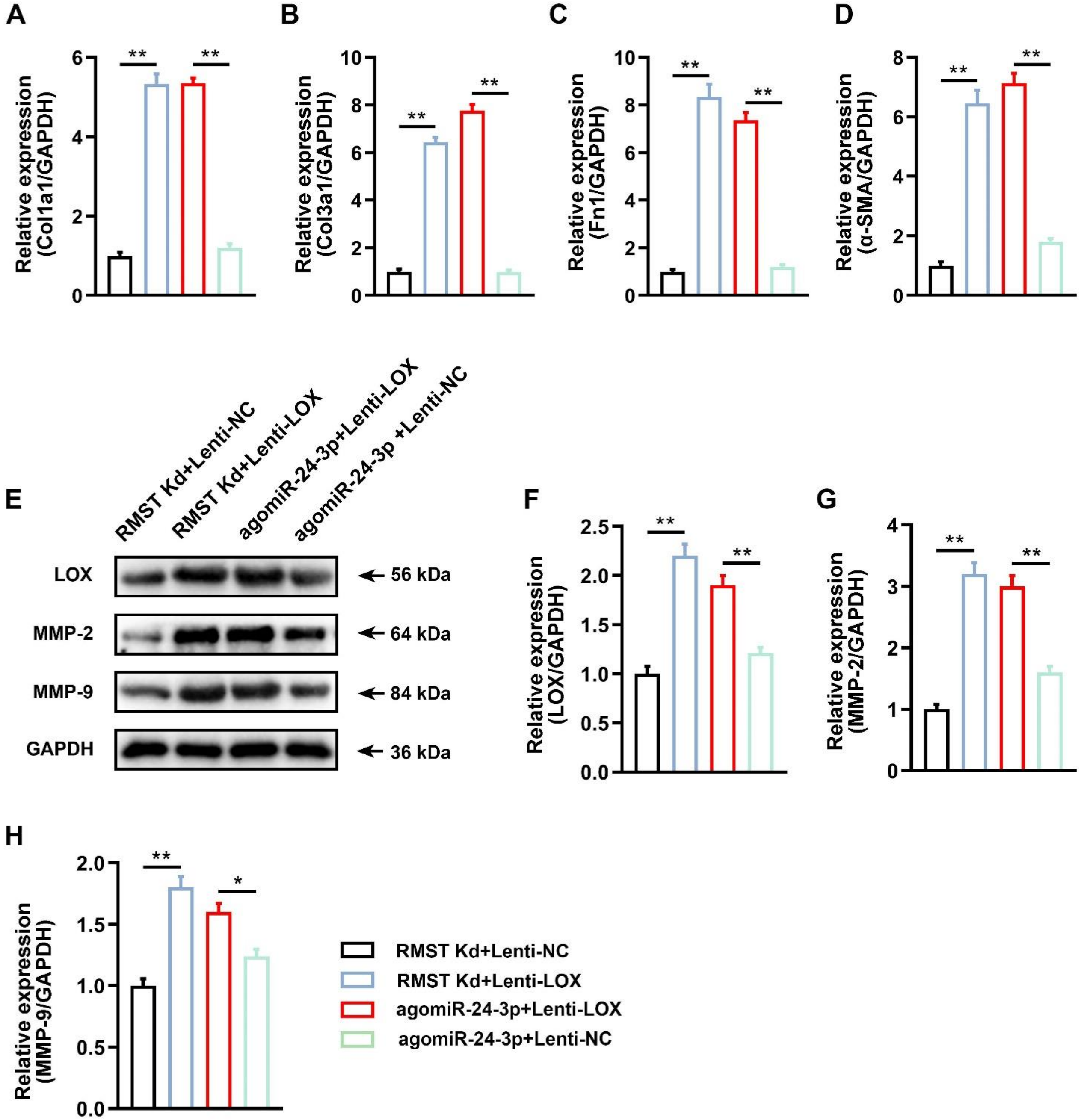

Figure S12. RMST regulates fibrotic-related protein expression through the LOX/MMP-2/MMP-9 pathway. (A–D) The mRNA expression of **(A)** Col1a, **(B)** Col3a1, **(C)** fibronectin, and **(D)** α-SMA in activated CFs was detected by qRT-PCR. **(E)** Representative images of protein expression of LOX, MMP-2, MMP-9, Col1a1, and Col1a3 detected by western blot. **(F–H)** Quantification of **(F)** LOX, **(G)** MMP-2, and **(H)** MMP-9 protein expression. $n = 4$ independent treatments. Significance was evaluated via one-way ANOVA with the post-hoc Bonferroni test. $^{**}P < 0.01$. LOX, lysyl oxidase; MMP-2, matrix metalloproteinase 2; MMP-9, matrix metalloproteinase 9; Col1a1, collagen type I alpha 1; and Col3a1, collagen type III alpha 1.

Figure S13

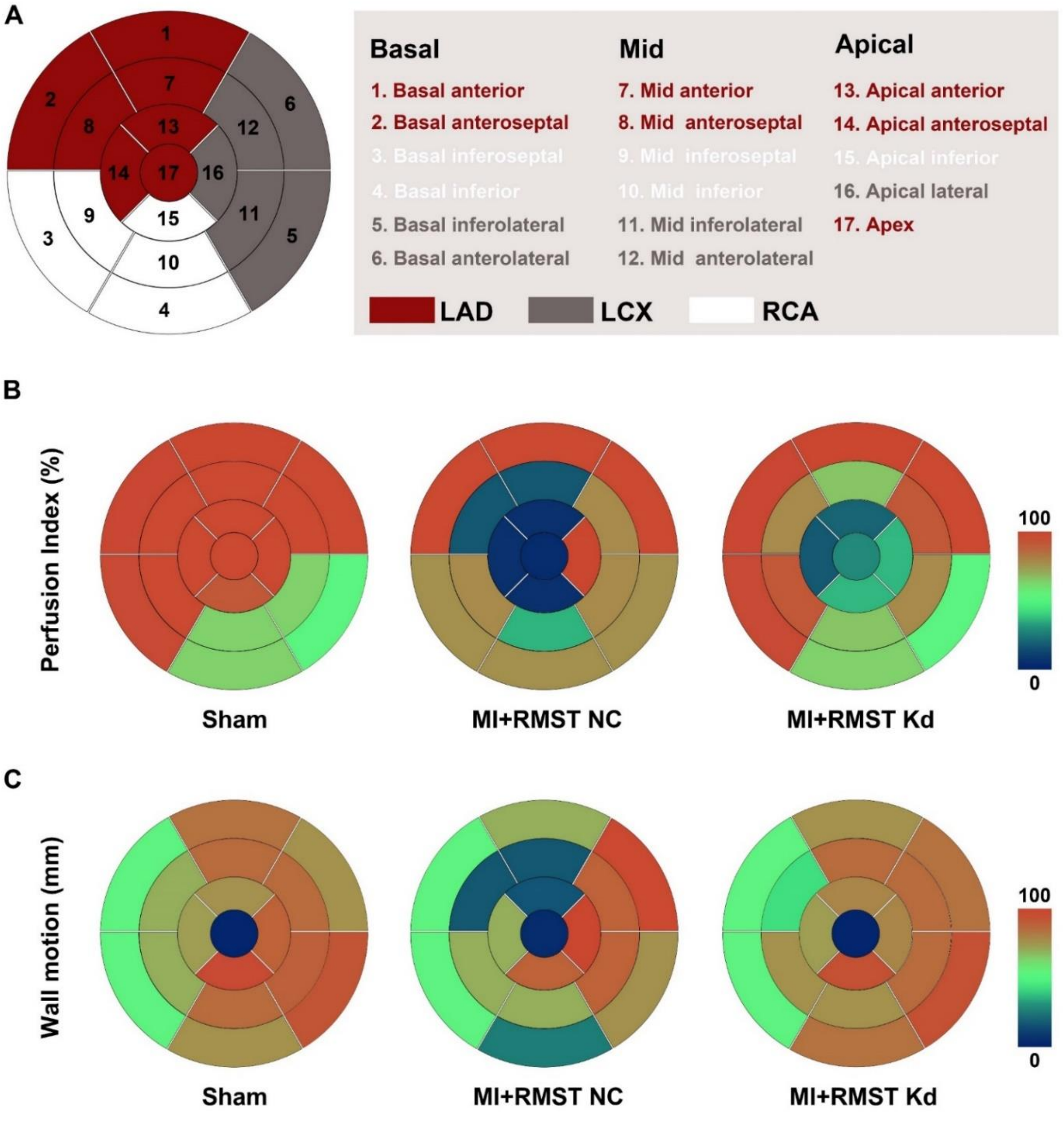

Figure S13. Heart perfusion and left ventricular wall motion analysis in pigs. (A) Schematic diagram of heart segments. **(B)** Heart perfusion is shown in the bull's eye diagram. **(C)** Left ventricular wall motion is shown in the bull's eye diagram. LAD, left anterior descending; LCX, left circumflex branch; and RCA, right coronary artery.

Table S1. The sequences of RMST shRNA

Species	Sequence (5’–3’)
Mouse	GGAGAGAACGGAATAGGACTCTGTACTCGAGTACAGAGTCCTATTCCG TTCTCTCC
Pig	TGATAGTGTGCTCTGTAATTACTCGAGTAATTACAGAGCACACTATCA

Table S2. The sequences of the primers

Gene Name	Sequence (5’- 3’)
**RMST (mouse)**	CCCTCCTCGAACTGAAAGATACTC
	GCCAAGTCTCCACGGTTCTATG
**RMST (pig)**	CCCGGAACAGGAAGCGATAG
	GGATTCTGCATGGCTGTAGTG
**miR-24-3p**	GCCGAGTGGC TCAGTTCAGC
	CTCAACTGG TGTCGTGGA
**LOX (mouse)**	TTCTTACCCAGCCGACCAAGATA
	GTGTTGGCATCAAGCAGGTCA
**LOX (pig)**	TCCAATGGGAGAACAACGGG
	GAGCCCCAGACGTCGAATAG
**Col1a1 (mouse)**	CATGTTCAGCTTTGTGGACCT
	GCAGCTGACTTCAGGGATGT
**Col1a1 (pig)**	CCAAGAGGAGGGCCAAGAAG
	CCAGCAGGACCAGCATCT
**Col3a1 (mouse)**	TCCCCTGGAATCTGTGAATC
	TGAGTCGAATTGGGGAGAAT
**Col3a1 (pig)**	GGGGCTCGAGGTAATGATGG
	GGTCGACCACTTTCTCCCTG
**Fn1 (mouse)**	CCTTCCTGTGGCTCCAGA T
	GCTGCCCCCATTCATACA
**Fn1 (pig)**	GATGAGCTTCCCCAACTGGT
	TTCCAGGAACTCGGAACTGC
**α-SMA (mouse)**	CCAGCACCATGAAGATCAAG
	TCCACATCTGCTGGAAGGTA
**α-SMA (pig)**	CCAGAGCAATCAGGGACC
	AGTTGGTGATGATGCCGTGT
**Myh7 (mouse)**	CGCATCAAGGAGCTCACC
	CTGCAGCCGCAGTAGGTT
**Myh7 (pig)**	GACCTGTCCCAGCTTCAGAC
	TCCAGGACTGGGAGCTTTGT
**Nppa (mouse)**	CACAGATCTGATGGATTTCAAGA
	CCTCATCTTCTACCGGCATC
**Nppa (pig)**	CAGCATGAGCTCCTTCACCA
	CTCCAAAAGGGGGCTGAGAG
**Nppb (mouse)**	GTCAGTCGTTTGGGCTGTAAC
	AGACCCAGGCAGAGTCAGAA
**Nppb (pig)**	AAGACGATGCGTGACTCTGG
	AGTTGCTTTGAAGGGGAGCA
**U6**	CTCGCTTCGGCAGCACA
	AACGCTTCACGAATTTGCGT
**GAPDH**	GCACCGTCAAGGCTGAGAAC
	TGGTGAAGACGCCAGTGGA

Table S3. List of the antibodies used in western blot

Primer antibodies	Source	Catalog#	Origin	Application
Col1a1	Abcam	ab270993	rabbit	1:1000
Col3a1	Abcam	ab2413	rabbit	1:1000
Ccnd1	Abcam	ab16663	rabbit	1:1000
Cdk1	Abcam	ab265590	rabbit	1:1000
LOX	Abcam	ab223488	goat	1:1000
MMP-2	Abcam	ab97779	rabbit	1:1000
MMP-9	Abcam	ab58803	mouse	1:1000
GAPDH	Abcam	ab8245	mouse	1:1000
β-tubulin	Abcam	ab15568	rabbit	1:1000
